# Grayscale projection two-photon lithography using sub-diffraction motifs for ultrafast and precise nanoscale 3D printing

**DOI:** 10.1038/s41467-026-73782-x

**Published:** 2026-05-29

**Authors:** Harnjoo Kim, Sourabh K. Saha

**Affiliations:** Scalable Technologies for Advanced Manufacturing Lab, George W. Woodruff School of Mechanical Engineering, Georgia Institute of Technology, Atlanta, GA USA

**Keywords:** Design, synthesis and processing, Lithography, Design, synthesis and processing

## Abstract

Rapid and high-fidelity nanoscale 3D printing is highly desirable, but it is difficult due to the tradeoff between speed and accuracy. Although optical projection techniques can massively scale up printing, fidelity is compromised due to the difficulty in precisely controlling the light dosage over the entire field. This challenge is typically addressed by using multiple projections, but it slows down printing. Here, we present grayscale projection two-photon lithography to overcome this tradeoff. Despite using a binary mask, it enables projecting more than 15,000 focal spots, each with independently tunable intensity. It advantageously leverages constraints imposed by optical diffraction to achieve grayscale tuning over the entire field at once. By directly tuning the focal spot intensities, we demonstrate suppression of proximity effects, compensation of non-uniform illumination, compensation of stitching artefacts, and rapid 3D printing with a single femtosecond pulse per layer. We demonstrate printing of nanowires as thin as 55 nm and achieve rates of 1.7 billion voxels/s and 215 mm^3^/hr.

## Abstract (web summary)

The ability to precisely tune light intensity within projected images can massively scale up sub-100 nm additive manufacturing. Kim and Saha show how optical diffraction can be leveraged to achieve this with a single pulse of femtosecond laser.

## Introduction

Rapid and accurate fabrication of mesoscale 3D structures with nanoscale features is highly desirable for creating nano-enabled devices that can address global challenges related to energy, healthcare, communications, computing, and sustainable development. Two-photon lithography (TPL) is a promising laser-based nanoscale 3D printing technique for creating polymeric 3D structures intended for real-world functional devices^1–5^. For example, complex nanostructured 3D structures fabricated via TPL have been demonstrated to be valuable in applications such as inertial fusion energy and plasma science^6–8^, micro-electro-mechanical systems (MEMS)^9,10^, micro-optics^11–16^, optical and mechanical metamaterials^17–20^, microsystems packaging^21^, and biotechnology^22,23^. Although TPL has been around for over two decades, it has remained a niche nanofabrication method limited to laboratory-scale use because of its slow processing speed^3,24^. Encouragingly, this limitation is becoming less severe now due to the development of various approaches that massively scale up the processing speed^25–32^. However, these scale-up approaches often lead to poorer fidelity or resolution due to the limited ability to deterministically tune the optical dosage at the high processing rates. Thus, a tradeoff exists between the rate of printing and the achievable fidelity. Here, we present the grayscale projection two-photon lithography (GP-TPL) technique that overcomes this tradeoff by enabling extensive optical dosage tunability in projection-based high-throughput TPL.

The ability to control the intensity of light with fine spatial and temporal resolutions is necessary to achieve high-fidelity printing with TPL. However, controllability often worsens when parallelization-based scale-up approaches are implemented. Traditionally, TPL is performed by focusing a beam of femtosecond laser into a focal spot inside a photopolymer resist material and serially scanning this spot^1,2^. The size of the printed volumetric pixel (i.e., the voxel) is determined by the intensity of the spot and the scanning speed. When high-fidelity printing is desired, the intensity must be modulated over multiple grayscale levels. While scanning a single beam, grayscale intensity control can be readily achieved with high-speed acousto-optic modulators^33,34^. However, this approach is not effective when multiple voxels are scanned at once. For example, when parallelization is achieved by diffractively splitting a single beam into multiple foci^27,32^, it becomes challenging to independently control the intensity of each focal spot. Holography-based parallelization schemes can natively control the intensity of multiple foci, but they do so by trading off geometric flexibility because the focal spots cannot be generated arbitrarily close to each other^25,29,31,35^. In principle, digital projection-based parallelization approaches can overcome these limitations by using liquid crystal-based spatial light modulators (SLMs) that enable pixel-level grayscale intensity control. However, such SLMs have low refresh rates and low damage thresholds, thereby making them impractical for patterning and projecting high-power femtosecond lasers. In contrast, micromirrors-based digital SLMs have high damage thresholds but often only allow for binary level (i.e., on versus off state) intensity control at each pixel^36^. Consequently, direct grayscale control of intensity has remained elusive in projection-based TPL.

In the absence of direct intensity-based grayscale control, projection-based TPL has evolved to implement grayscale control via tuning of exposure time. However, the inability to independently tune the intensity of the projected focal spots introduces a severe rate versus accuracy tradeoff in projection-based TPL. As the dosage required for printing is highly sensitive to the proximity of features, projections that contain both densely and sparsely packed features are susceptible to defects. Specifically, proximity-based defects manifest as excessive uncontrolled polymerization in the denser regions and under-polymerization in the sparser regions^26,37^. Proximity effects are well known in TPL^38–41^, and these effects are generally suppressed in layer-by-layer processing approaches by averaging exposures over time^26,28,37,39^. For example, we have previously demonstrated how the proximity effects can be minimized by projecting a sequence of different images over time^26^. This approach is conceptually similar to the duty-cycle-based grayscale techniques that generate uniform dosages by averaging the light exposure over time. Such techniques are widely used to control the dosage in projection-based single-photon stereolithography techniques that use binary digital masks^42–45^. However, these exposure time-based grayscale approaches are effective only when multiple pulses of femtosecond laser are used to write each layer because they rely on modulating the duration of exposure (i.e., equivalent to the number of pulses). Thus, they slow down printing and make it impossible to print each layer using only a single femtosecond pulse, which corresponds to the highest rate that is theoretically achievable with layer-by-layer TPL techniques. Holography-based grayscale techniques that can natively control the intensity of each individual focal spot also require multiple pulses to write a single layer as the focal spots must be scanned within each layer^29,31^. Thus, existing grayscale approaches are slow due to their inability to write entire layers using only a single femtosecond pulse.

Additionally, time-averaging grayscale techniques attempt to indirectly compensate the effect of non-uniform intensity by generating spatially non-uniform durations of exposure. In principle, this approach can achieve a uniform dosage; however, it is challenging to do so in practice because the scaling of dosage with intensity and duration of exposure varies widely with material systems and proximity^6,37–39^. Thus, to achieve high-fidelity printing, one must perform extensive guesswork-based iterative experiments to identify the appropriate mask images. These problems become exacerbated if the incoming beam itself has a non-uniform intensity distribution, such as a Gaussian profile. Furthermore, due to the nonlinear scaling of dosage with intensity, compensating for small variations in intensity requires large variations in exposure time, which massively slows down printing. Our GP-TPL technique overcomes these challenges by providing the means to individually tune the intensity of each individual focal spot within the same projection even with binary micromirror-based SLMs. Furthermore, it enables achieving this tuning with a single pulse of femtosecond light, thereby enabling massive scale-up of high-fidelity TPL while maintaining process controllability.

## Results

**Fig. 1:**
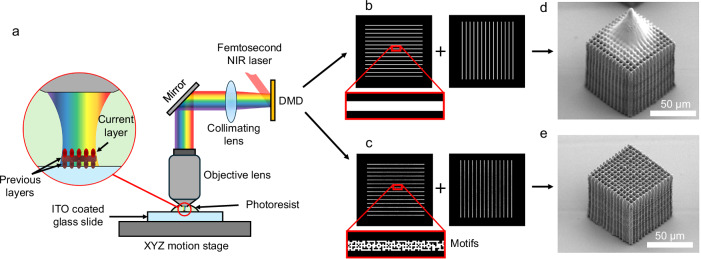
Grayscale projection two-photon lithography using sub-diffraction motifs. **a** Schematic of the projection-based two-photon lithography system. Figure adapted with permission from Kim et al.^61^, Copyright 2022 ASME. **b** Bitmap images of fully white grating lines used for projection. **c** Bitmap images of grating lines composed of motifs used for grayscale projection. **d** Exemplary structure with localized over-polymerization, printed by projecting fully white grating lines and with a Gaussian beam. **e** Exemplary structure printed with motif-based lines, without localized over-polymerization despite being exposed to a Gaussian beam.

Our GP-TPL technique is based on the principle of spatio-temporal focusing of a patterned beam of femtosecond (fs) laser. The beam is patterned with a digital micromirror device (DMD), which acts as a binary intensity mask. The mask is programmed to display a specific pattern of on-state and off-state micromirrors by loading a 1-bit bitmap image. The on-state micromirrors redirect the incident fs laser light toward the collimating lens, whereas the off-state micromirrors redirect the light out of the projector. Thus, the DMD generates a patterned beam of fs laser. This patterned beam is then focused into the photoresist using a set of collimating and objective lenses, as illustrated in Fig. 1a. The lenses are arranged in a 4f-like system to ensure spatio-temporal focusing. Spatio-temporal focusing enables projecting a patterned light sheet, comprising thousands of focal spots, within the interior of the photopolymer resist and applying it to cure a thin section without curing any material below or above the focal plane^26,28,46^. It is achieved by temporally stretching and compressing a femtosecond pulse as it propagates through the photoresist material. Spatio-temporal focusing originated in the field of parallel two-photon microscopy^47–49^, and we have previously applied it to scale-up various two-photon absorption-based fabrication processes such as polymer 3D printing^26,37^, metal printing^50^, metal sintering^51^, and metal ablation^52^. Our GP-TPL technique fundamentally advances the capability of projection TPL by enabling the deterministic tuning of the intensity of each focal spot within the spatio-temporally focused light sheet.

GP-TPL leverages the diffraction limit of light to achieve grayscale tuning of intensity using binary digital masks. Specifically, it achieves this by projecting a pre-determined binary motif pixel pattern for each individual focal spot. All motif patterns have the same number of total pixels (i.e., sum of on and off pixels), but each motif corresponds to a specific intensity level from 100% to 10%. Thus, when a particular non-uniform intensity distribution is desired, it is achieved by projecting a binary image comprising various motif patterns. For example, non-uniform over-printing can be effectively compensated by projecting images containing various motifs, as illustrated in Fig. 1b–e.

At its core, GP-TPL leverages these two scientific principles: (1) light cannot be focused into arbitrarily small spots due to the diffraction limit imposed by the focusing optics and (2) the amount of light in a projected focal spot can be reduced to arbitrarily small values by switching off pixels within that spot. In combination, these two principles enable reducing the optical power of a focal spot without reducing its size, thereby enabling the tuning of intensity. We have observed that to be able to effectively apply these principles for intensity tuning of focal spots, the size of the motif pattern should not deviate significantly from the diffraction-limited focal spot size of the optical system. This spot size can be estimated from the diameter of the first Airy disk that is formed when a Gaussian beam of the same center wavelength is focused using the same objective lens as those present in the projection system. For our system, this diameter is equal to 785 nm (i.e., 1.22λ/NA = 1.22 × 804 nm/1.25). As the demagnified width of each pixel at the focal plane is 114 nm (i.e., pixel pitch of DMD times optical magnification), 7 pixels can fit within the width of a diffraction-limited spot. Motifs wider than the diffraction limit produce multiple intensity peaks and complex shapes, while much smaller motifs, such as 5-pixel wide and 3-pixel wide, significantly reduce intensity (as illustrated in Supplementary Figs. S1, S2). In contrast, motifs on the scale of the diffraction limit (i.e., 7-pixel wide) can generate nearly Gaussian intensity distributions with intensities within 10–100%. Thus, we have selected square motifs of side length 7-pixel to achieve grayscale control of intensity.

**Fig. 2:**
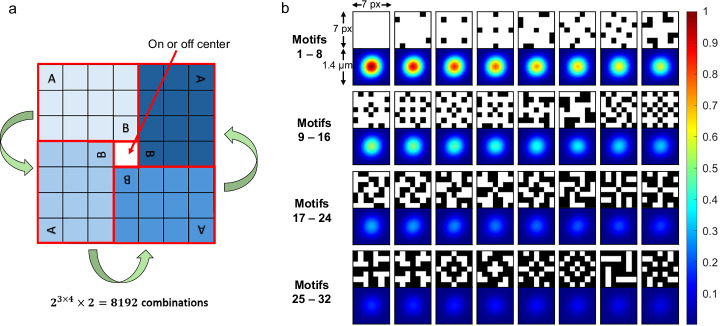
Sub-diffraction motifs used for grayscale projection two-photon lithography. **a** Schematic of motif generation. Each square box in the motif pattern represents one pixel, i.e., one micromirror in the DMD. **b** List of 32 binary motifs of size 7×7 pixels and the corresponding simulated intensity profile for each motif. Intensities are normalized to the peak intensity of the first motif.

As there are a total of 2^49^ (i.e., half a quadrillion) distinct motifs of size 7×7 pixels, the selection and identification of the relevant motifs is non-trivial via experimentation or simulation. Therefore, we have narrowed down the motifs by applying fourth-order rotational symmetry, as shown in Fig. 2a. Within the 7×7 footprint, we select a 3×4 sub-pattern which is then rotated and replicated four times over the 7×7 region. This leaves the central point to be either on or off for each sub-pattern. Our strategy reduces the number of unique motifs to 2^13^ (i.e., 8192 combinations), which leads to a smaller and more tractable set of motifs. Over this set, we have performed physics-based simulations and experiments to determine the relationship between the motif pattern and its intensity distribution. Based on these data, we refined our selection to a set of 2^5^ (i.e., 32) motifs that have the same nominal full-width-half-maximum (FWHM) diameter, but with peak intensities varying from 100% to 10%. The motif with 100% intensity is the one in which all 49 pixels are on. The 32 motifs and the corresponding simulated intensity distributions are shown in Fig. 2b. We selected the 32 motifs by first identifying those motifs that had the same nominal simulated FWHM size as the fully white motif and then choosing the motifs that progressively reduced the intensity by ~7.7% relative to the intensity of the previous level. The details of the motif selection process are illustrated in Supplementary Figs. S3–S7.

We have experimentally verified that the focal spots of the 32 motifs are nominally of the same size. The average FWHM diameter of the 32 motifs was measured to be 830 nm, and the 1-σ standard deviation of the FWHM was 14 nm. The experimental FWHM is larger than the simulated FWHM by 40%. We believe that the discrepancy between the simulated and the experimental FWHM is due to optical aberrations in the system, which were not captured in the simulations. This is supported by our observation that the average measured FWHM value changed by as much as 100 nm after major realignments of the projector, but the 1-σ standard deviation of the FWHM diameters of the various motifs remained bounded (1-σ < 15 nm). Thus, despite the aberrations, the motifs generate focal spots of the same nominal size. It is noteworthy that in all 32 motifs, there is at least one bright pixel at each edge of the 7×7 square pattern. Motifs that are smaller, such as patterns in which all on pixels fit within the 5×5-pixel footprint, generally have a smaller FWHM. Furthermore, motifs in which the on pixels are concentrated primarily at the edges have a higher FWHM. Additional data on the measured and simulated FWHM size of the motifs are summarized in the Supplementary Figs. S5–S7.

**Fig. 3:**
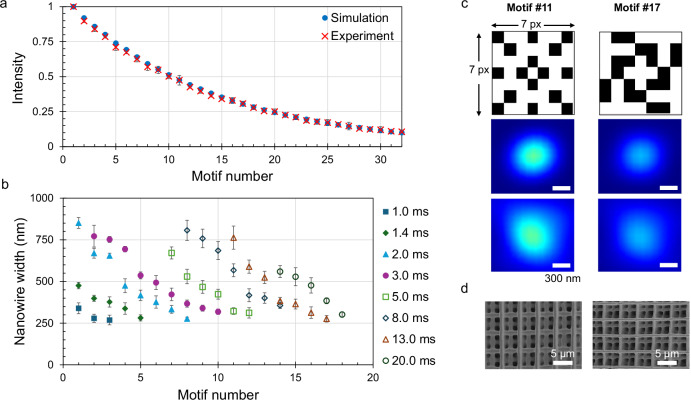
Deterministic tuning of voxel-level intensity and voxel size via sub-diffraction motifs. **a** Simulated and measured relative peak intensity of each motif versus motif number for the 32 motifs of size 7×7 pixels. Each experimental datapoint represents the mean from 5 measurements of the same motif pattern located at different positions within a single projection. Error bars indicate 2-σ standard deviation. **b** Width of nanowires in 3D woodpiles printed with a single motif versus the motif number for various exposure times. Each datapoint represents the mean from 5 measurements and the error bars indicate 2-σ standard deviation. **c** Two exemplary motifs and their intensity profiles (top row: from simulations, bottom row: from experiments). Scale bars are 300 nm long for all profiles. **d** Exemplary woodpile structures printed with motifs 11 (left) and 17 (right) with 13 ms exposure time.

The intensities of the motifs follow a predictable trend and the simulated and experimental peak intensities match well, as shown in Fig. 3a. Thus, these motifs enable deterministic tuning of the intensity of each focal spot over 2^5^ levels, while holding a constant focal spot size. We have verified that this intensity tunability does indeed translate into the practical capability of controlling the size of the printed voxels. For these tests, we printed 3D woodpile structures by projecting the same motif over the entire projection, while holding the beam power constant. It was observed that by changing the motif, the linewidth could be varied by hundreds of nm. For example, while holding the exposure time at 2 ms, the linewidth can be varied from 850 nm to 275 nm by changing the motif. Additionally, as expected, woodpiles could not be produced with lower durations of exposure and higher numbered motifs due to the low optical dosage under such conditions. The data for these tests are summarized in Fig. 3b and additional images of the printed woodpiles are shown in the Supplementary Fig. S8. For each exposure time, varying the motif enables deterministically tuning the width of the nanowires. Therefore, these motifs replicate the voxel-level grayscale capability of serial point-by-point TPL, while simultaneously scaling up printing via such massive parallelization that is not available in serial TPL.

**Fig. 4:**
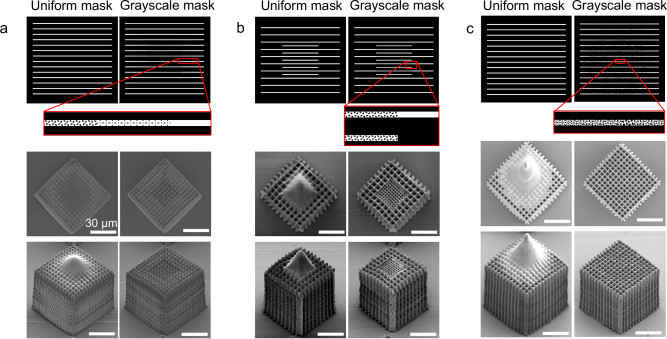
Grayscale compensation of defects arising from proximity effects and non-uniform illumination. **a** Compensation of over-polymerization in the center of woodpiles arising from proximity effects. Grayscale mask contained motifs of three different intensities: 100%, 80%, and 60%. **b** Compensation of over-polymerization in the region of higher feature density arising from proximity effects. Grayscale mask contained two types of motifs: 100% in sparser and 60% in denser region. **c** Compensation of over-polymerization due to non-uniform illumination arising from the Gaussian beam profile. Grayscale mask contained the first 17 motifs with intensities varying from 100% to 31%. In each sub-part (**a**–**c**), left columns show masks and woodpiles for uncompensated printing with fully bright masks whereas right columns show masks and woodpiles for compensated printing with grayscale masks. Bottom rows show 45-degree tilted view of the same woodpile shown in the middle row. Scale bars are 30 μm.

We have leveraged the grayscale capability of motifs to compensate for non-uniform printing that arises from proximity effects and non-uniform beam profiles. These compensations are illustrated in Fig. 4. The left column in each panel shows the control bitmap mask pattern with fully bright motifs and the corresponding printing outcomes, whereas the right column shows the patterns with grayscale motifs and the corresponding outcomes. For each panel, the control and compensated masks were projected with the same beam power. For panels (a) and (b), the exposure time was held constant; for panel (c), grayscale printing was performed with a higher exposure (3 ms versus 2 ms). As illustrated in Fig. 4a, excessive uncontrolled printing can occur in the central region, which is the densest region of the projection due to its proximity to other features. These proximity-based defects have also been observed in other parallel TPL implementations^29,39^, and they originate from the spatiotemporal dynamics of the underlying photopolymerization reactions. In the compensated projections, three different 7×7 motifs have been used. The motifs around the boundary are fully bright motifs that correspond to 100% intensity, those in the central region correspond to 60% intensity, and those in the intermediate region correspond to 80% intensity. The printing outcomes for the two sets of masks exhibit extensive differences in the quality of printing. Distinct features were not observed in the central region of the uncompensated woodpile due to over-printing whereas distinct features without over-printing were observed in the central region of the compensated woodpile. We have also tested the effectiveness of the grayscale masks in suppressing the proximity effects in structures printed with resists of various optical sensitivities. Although the exposure time at which the proximity effects became prominent varied with the sensitivity of the resist, we observed that the proximity effects could be suppressed with the same set of masks. These results are summarized in the Supplementary Fig. S17. This observation is consistent with our past work wherein we demonstrated that the proximity effects arise due to the low sparsity of features in the images^37^. Thus, the same set of masks are effective in suppressing the proximity effects with various resists because the sparsity in the images remains unchanged when the sensitivity of the resist is changed.

Proximity-based defects become exacerbated in non-uniform structures that have large differences in feature densities, and our grayscale technique can compensate even for these defects. For example, the structure on the left column of Fig. 4b exhibits over-printing defects because it contains regions with two different feature densities, i.e., regions with 56-pixel period and 28-pixel period. It has been previously observed that the over-printing defects emerge at a lower dosage when the period of the structures is lower^37,39^. Thus, dosages that may be optimal for the 56-pixel period may lead to over-printing for the 28-pixel period. Therefore, structures having two or more periods are challenging to print via projection-based TPL because the optimal dosage window for each period may have minimal overlap. For example, at low dosage, over-printing may be absent in the 28-pixel regions, but under-printing may occur in the 56-pixel region. Conversely, at high dosage, optimal printing may occur in the 56-pixel region, but over-printing may occur in the 26-pixel region. In the past, we have demonstrated how this challenge can be solved by splitting the original image into multiple sparse images and then projecting them in a sequence^37^. However, this approach slows down printing. Our grayscale technique provides a faster compensation mechanism. By applying a 60% intensity motif in the denser region, the over-printing defect was fully eliminated with a single mask image. Thus, motifs can effectively compensate for the defects arising from a broad set of proximity effects while preserving the high throughput of projection-based TPL.

Our grayscale technique can also compensate for non-uniform intensity profiles in the beam. Ideally, the beam should have a flat profile so that a uniform light intensity can be generated over the entire projection. However, this ideal uniform condition is difficult to achieve in practice due to a combination of misalignment in the beam flattening optics, asymmetric beam profile originating from the laser, and dusty or damaged optics. The local intensity peaks in the non-uniform beam profile can cause localized over-printing defects within the projected area. Here, we have demonstrated the ability to compensate for these defects by purposely printing with a non-uniform Gaussian beam by removing the beam shaper from the optical path. Doing so causes the intensity to vary over the area of projection by a factor of 3.3 times. As shown in Fig. 4c, the non-uniform beam leads to over-printing near the top-corner of the woodpile, which corresponds to the region of the beam with the highest intensity. The high-intensity region of the beam was purposely offset from the center of the projected image to highlight the effect of non-uniform intensity, which is distinct from the effect of proximity effects that cause over-printing at the center of the projection. To eliminate the defect arising from beam non-uniformity, the intensity profile of the beam was first measured with a beam profiler and then a mask image was produced for compensation by applying lower intensity motifs in the regions of higher intensity. This resulted in a mask with smoothly varying motifs. The details of the compensation technique are shown in the Supplementary Figs. S11–13. As illustrated in Fig. 4c, upon application of the compensated mask, the over-printing due to non-uniform Gaussian beam was eliminated. Thus, our work demonstrates the effectiveness of the motif-based grayscale technique in compensating beam non-uniformity using a single mask image.

**Fig. 5:**
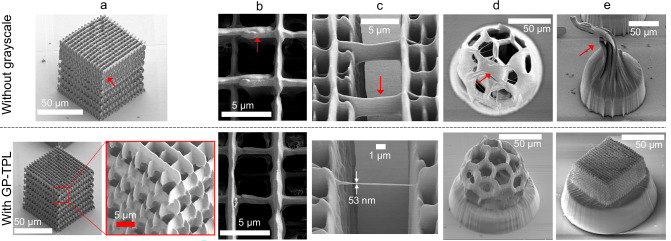
Ultrafast and precise nanoscale 3D printing enabled by grayscale projection two-photon lithography (GP-TPL). **a** Woodpiles printed with fully bright motifs exhibiting blocked pores on upper layers (top) and with grayscale masks exhibiting porosity along the entire vertical surface (bottom). **b** Voxel-level lap joints printed by partially overlapping fully white motifs along the horizontal axis (top) versus lap joints printed with grayscale motifs in the overlap zone (bottom). **c** Suspended wires printed with fully bright motifs (top) and a sub-100 nm nanowire printed with grayscale motifs (bottom). Marked width was measured from a separate top-down view. **d** Buckyball structures printed with fully bright motifs (top, over-polymerized) versus grayscale motif number 15 (bottom, optimal). **e** Excessively over-polymerized solid cylinder printed with fully bright motifs (top) and porous lattice printed on top of an optimally polymerized solid cylinder with grayscale motifs (bottom). Continual axial scanning with single femtosecond pulse per layer was used to print all panels in (**d**) and (**e**).

To further demonstrate the versatility of GP-TPL in improving the fidelity of printing, we have printed exemplary woodpile structures shown in Fig. 5a. Proximity effects may not only lead to merging of features within a single layer (as demonstrated in Fig. 4a) but may also cause features from different layers to merge along the height. For example, when fully white motifs were used to print a porous 3D woodpile, several of the upper layers merged, thereby closing the pores along the vertical surfaces (Fig. 5a, top panel). We compensated for this effect by using grayscale masks in each layer and printing all layers with the same exposure time. As shown in the bottom panel of Fig. 5a, porosity is maintained over the entire height of the vertical surfaces as the features from the different layers do not fully merge. We have also applied GP-TPL to implement voxel-level lap joints that are analogous to lap joints in woodwork. These joints may be implemented to stitch together multiple fields and produce structures that are larger than the area of a single projection. However, lap joints are challenging to produce with TPL due to a combination of misalignment errors from the motion stages and excessive printing near the joints^6,26^. Out of these two sources, GP-TPL can suppress the over-printing defects during field stitching. To decouple the effect of motion errors, we performed side-by-side printing of two smaller projections that fit within the same field. The projections were identical in architecture and were partially overlapped along the horizontal direction (as illustrated in Supplementary Fig. S25). As expected, when fully white motifs were used, excessive voxel broadening was observed in the overlap region (Fig. 5b, top). In contrast, with motifs of lower intensity, the voxel broadening in the overlap region was significantly suppressed (Fig. 5b, bottom). We envision that the voxel-level dosage tunability of GP-TPL can be applied to improve the fidelity of printing in a variety of applications.

The ability of GP-TPL to precisely tune the intensity at each individual focal spot enabled us to push the minimum feature size resolution into the sub-100 nm regime. As shown in Fig. 5c, printing with 7×7 fully white motifs produced wires that were narrower than 300 nm along the width but were more than 1 μm along the height. After applying the motifs, the width and height reduced to as much as 53 nm and 112 nm, respectively. These nanowire dimensions correspond to a voxel size of 83 nm based on the arithmetic mean of width and height and a voxel size of 77 nm based on the geometric mean. All processing parameters, other than the mask images, were held constant during printing of the wires shown in the top versus the bottom panel of Fig. 5c. It is noteworthy that GP-TPL can reduce the linewidth to less than 100 nm even in regions near the anchor points, such as the right edge in the lower panel of Fig. 5c. Additionally, the thinnest section of the nanowire corresponds to the motif of the lowest intensity. Details of the compensation mask and dimensions along the length are shown in Supplementary Fig. S26. Maintaining fine linewidths is challenging near anchor points due to the accumulation of dosage from the support pillars. GP-TPL enables compensating for this non-uniformity of dosage and thereby achieve fine control of the linewidth.

In addition to improving the quality and resolution of printing, GP-TPL massively increases the rate of printing by enabling high-fidelity printing with single femtosecond (fs) pulses. Specifically, it enables continuously scanning the focal plane along the depth (i.e., axial) direction while printing each layer using a single pulse of fs laser. This contrasts with the approach of layer-by-layer printing wherein each layer is printed by projecting multiple pulses and the motion stage must stop and settle at each layer before the beginning of projection. The continual axial scanning approach scales up printing by eliminating non-productive time during stage stop-and-settle cycles. In the axial scanning approach, the rate can be maximized by printing each layer with a single fs pulse and by scanning at the repetition rate of the laser (i.e., at a speed of 5 kHz per layer for our system). However, practically achieving this printing modality is challenging and past implementations of continual axial scanning projection TPL have failed to achieve printing with a single fs pulse per layer^28^. A key barrier in achieving this is that when a single pulse of fs laser is used, it collapses the two-dimensional input parameter space for dosage control, which is spanned by intensity and exposure time, into a smaller unidimensional space. This is so because the exposure time is now fixed (to the duration of a single pulse) and it cannot be exploited as a controllable input parameter anymore. This limitation makes it challenging to finely control and optimize the dosage over the entire field of projection to achieve sufficient curing at the boundaries without over-printing in the center. In our tests, we observed significant over-printing defects in the central region, which were eliminated by applying grayscale masks comprising various motifs. These printing outcomes are illustrated in Fig. 5d, e. Additional details on the axial-scanning printing modality are shown in Supplementary Figs. S18–21. During printing of solid cylindrical bases without grayscale masks (Fig. 5e, top), the over-polymerization is so extreme that it prevents stacking any other structure on top of the base. In contrast, GP-TPL enables suppressing these defects and rapidly printing complex 3D structures of various geometries, such as a lattice structure on top of a solid cylinder (Fig. 5e, bottom). These tests demonstrate the printing of 3D structures via continual axial scanning projection TPL and with a single fs pulse per layer.

**Fig. 6:**
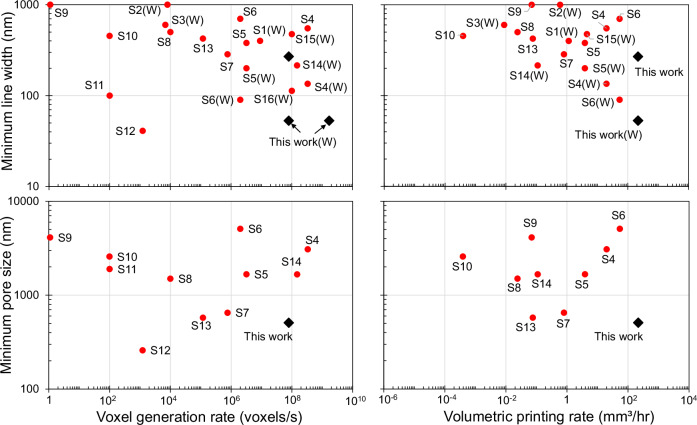
Comparison of resolution versus rate of grayscale projection two-photon lithography (GP-TPL) with the state-of-art two-photon lithography techniques. Studies include: S1^27^, S2^53^, S3^54^, S4^26^, S5^28^, S6^29^, S7^37^, S8^25^, S9^55^, S10^56^, S11^19^, S12^57^, S13^6^, S14^31^, S15^32^, S16^58^. Metrics were determined for printing of isolated nanowires (indicated by study markers with W in parenthesis) and porous 3D structures.

We have benchmarked the printing rate and resolution of GP-TPL with those of past TPL implementations^6,19,25–29,31,32,37,53–58^, as shown in Fig. 6. The rate was quantified in terms of the voxel generation rate and the volumetric printing rate. The resolution was quantified in terms of the width of the finest printable feature and the width of the smallest printable pore. With continual axial scanning, we have achieved printing of each layer using a single pulse and at a rate equal to the repetition rate of the laser, i.e., at 5 kHz. As demonstrated in Fig. 5e, the maximum area of each layer corresponds to a circle with a diameter of 113 μm (i.e., equivalent to 994 pixels). As the axial spacing between the layers is 1.2 μm, our volumetric printing rate is 217 mm^3^/hr. This rate is higher than that of any other TPL technique that can make sub-micron features (top-right panel of Fig. 6). Furthermore, no other high-throughput TPL technique has been able to print nanowires as narrow as 53 nm. The data for Fig. 6 are listed in the Supplementary Table S4. It is noteworthy that a distinct whitespace exists in the region of sub-micron pores and high throughput (bottom-right panel of Fig. 6). This is unsurprising because it is extremely challenging to fabricate such nanoporous 3D structures due to the proximity effects inherent to TPL. GP-TPL excels in producing nanoporous 3D structures rapidly, and it is two to three orders of magnitude faster that than other TPL implementations with comparable porosity. For example, it is 270 times faster than our past demonstration of interspersing-based projection TPL (study S7^37^) and three thousand times faster than high-speed serial TPL (study S13^6^).

The voxel generation rate of GP-TPL is comparable to or exceeds the rate of past high-throughput TPL implementations. As the largest circle that can be projected has a diameter of 994 pixels and each focal spot is generated by motifs that are 7×7 pixels wide, a total of 15,836 focal spots can fit within the area of each projection. If we consider that each focal spot maps to a single voxel, then the peak voxel generation rate of GP-TPL is 7.9 ×10^7^ voxels/s (i.e., 15,836 voxels ×5 kHz). However, if we consider that each voxel is as big as the demonstrated minimum feature size (i.e., 77 nm for minimum voxel size based on geometric mean of width and height), then 107 voxels fit within the footprint of each 7×7-pixel motif. As the nanowire was printed with 5 pulses, it corresponds to a voxel generation rate of 1.7 ×10^9^ voxels/s (i.e., 107 voxels per focal spot × 15,836 focal spots × 5 kHz / 5 pulses). We have reported both these rates in Fig. 6. Depending on which definition is being used, the voxel generation rate of GP-TPL is comparable to or exceeds the rate of past high-throughput TPL implementations, including those based on holography (study S6^29^, S14^31^), projection TPL (S4^26^), axial-scanning projection TPL (S5^28^), multi-beam serial scanning (S1^27^, S15^32^), and scanning with metalens arrays^58^. Furthermore, amongst techniques capable of printing nanowires with sub-60 nm linewidths, the voxel generation rate of GP-TPL is at least 0.6 million times higher (i.e., GP-TPL vs. study S12^57^). Thus, GP-TPL provides a rapid nanoscale 3D printing capability for high-fidelity printing of nanoporous 3D structures with fine nanoscale features.

**Fig. 7:**
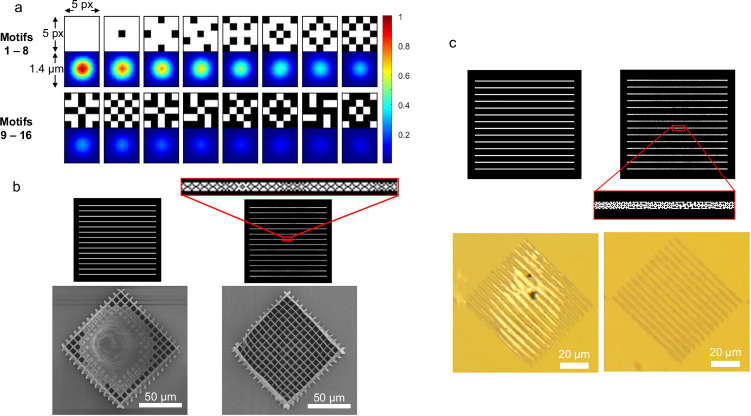
Generalizability of grayscale projection two-photon lithography (GP-TPL) and the motif-based grayscale approach. **a** Designs and simulated intensity distributions of the 5×5-pixel motifs generated for the 40× 1.3 NA objective lens. **b** Compensation of non-uniform illumination with a Gaussian beam profile during GP-TPL-based polymer printing. SEM images of woodpile polymer structures printed with fully bright masks (left, bottom) and compensated grayscale masks (right, bottom). **c** Compensation of non-uniform illumination with a Gaussian beam profile during motif-based grayscale metal ablation of gold thin film, using 60× 1.25 NA objective lens. Optical images of line arrays ablated with fully bright mask (left, bottom) and grayscale mask (right, bottom). Brighter regions indicate excessive removal of the gold film in regions of high intensity.

So far, we have demonstrated the capabilities of GP-TPL using a specific objective lens and the two-photon polymerization process, but the motif-based grayscale approach can be broadly generalized to be effective with other lenses and processes. To implement GP-TPL with other optical configurations, such as a different objective lens or a DMD, one must first quantify the characteristic lengths of the projection optics. These lengths are captured by the pixel pitch in the image and the diffraction-limited spot size of the objective lens. If the number of pixels that fit within the footprint of the diffraction-limited spot remains unchanged (i.e., equal to 7 pixels) in the new configuration, then we anticipate that the motifs shown in Fig. 2 will not require recalibration. However, recalibration of motifs will be necessary if the number of pixels changes. For example, with a different oil immersion objective lens (40× instead of 60× magnification and a numerical aperture of 1.3 instead of 1.25), each pixel is 171 nm wide and the diameter of the diffraction-limited spot is 755 nm. With these characteristic lengths, the diffraction-limited spot is no more than 5 pixels wide. Thus, the motifs should be 5×5 pixels wide, which leads to a set of 2^25^ motifs. Owing to the smaller footprint, the motif designs are expected to be different from those shown in Fig. 2. Despite this, we successfully identified distinct motifs that deterministically tune the intensity of the focal spots. Through fourth-order rotational symmetry considerations, we reduced the entire set of 2^25^ motifs to a set of 2^7^ motifs. Through simulations and experiments, we further refined this to a set of 2^4^ (i.e., 16) motifs that can tune the intensity of the focal spot from 10 to 100%, while holding the spot size nearly constant. Furthermore, we demonstrated that these motifs can be applied to compensate and overcome the over-printing defects arising from a non-uniform Gaussian beam profile. The generalizability of GP-TPL is summarized in Fig. 7. Additional data for the 40× objective lens are available in the Supplementary Figs. S14, 15. Thus, GP-TPL is broadly generalizable as it is not limited to just a unique combination of lenses.

As the motif-based grayscale approach does not rely on process-specific dosage laws to achieve the grayscale effect, it can be broadly applied to achieve voxel-level intensity control even in other femtosecond laser processing techniques, such as ablation-based micromachining. As an exemplary demonstration of the generalizability of the motif-based approach, we have performed micromachining of thin gold films with the 60× objective lens and with a non-uniform Gaussian beam. Details of the demonstration are shown in Fig. 7c and the Supplementary Fig. S16. Without mask compensations, non-uniform ablation is observed wherein the patterns at the edges are partially ablated whereas those in the center are excessively ablated. Applying the motif-based compensated mask led to uniform ablation over the entire area of projection. It can be envisioned that the grayscale intensity control capability of the motifs can be broadly applied to tune the intensity field of projected fs light sheets for a variety of fabrication processes such as projection-based sintering and metal printing.

## Discussion

Here, we discuss the physical basis of GP-TPL from the perspective of predictively generating the palette of motif patterns for desired grayscale levels. In general, motifs with higher intensities have a larger number of ‘on’ pixels, as evidenced by the larger number of ‘on’ pixels in the motifs on the top row versus the bottom row of Fig. 2a. Based on this observation, one may hypothesize that the intensity of each motif can be predicted from the weighted sum of intensity contributions of each pixel, wherein the contributions are weighted by the distance from the center using a Gaussian distribution. Upon testing this hypothesis, we found that this simplistic approach cannot accurately predict the intensity or the ordering of the motifs, as illustrated in the Supplementary Fig. S22. To accurately predict the ordering, one must also account for the spatial frequency information encoded in the motif patterns. Due to its finite sized aperture, the objective lens acts as a spatial frequency filter and it blocks out higher spatial frequencies from reaching the focal plane. In the past, this spatial filtering effect was leveraged to develop a grayscale projection approach by tuning the spatial frequency on a length scale much larger than the diffraction-limited light spot^59^. Our work advances the state-of-art by demonstrating that the spatial filtering effects can be effective even at sub-diffraction length scales and that the intensity of each individual focal spot can be deterministically tuned over numerous levels. Our physics-based simulation of the intensity of the motif patterns is based on Fourier optics techniques, which inherently incorporate spatial filtering through Fourier transforms. Thus, our simulations can be applied to accurately predict and select the motifs for the desired grayscale levels.

In summary, we have presented a grayscale projection two-photon lithography (GP-TPL) technique that enables deterministic and independent tuning of the intensity of numerous focal spots simultaneously and over multiple discrete levels. This was achieved by projecting sub-diffraction binary motif patterns for each focal spot. By applying the motifs, the intensity profile over an entire 2D projected area can be deterministically tuned to overcome various processing defects. For example, defects due to proximity effects and non-uniform illumination were overcome by using binary images comprising various motifs. GP-TPL is broadly generalizable to projection systems with different lens parameters and to other processes in which a patterned beam is projected. The key to enabling grayscale control of intensity is to ensure that the magnification and numerical aperture of the projection system are such that multiple pixels can fit within the diffraction limit. Specific motifs can then be identified by applying symmetry principles and turning some of the pixels off within the motif. The specific relationship between the motif pattern and the intensity can be predicted from physics-based simulation of the intensity profile. Here, we have demonstrated printing of nanoporous 3D structures with fine pores at rates that are at least two orders of magnitude higher than the state-of-art. Furthermore, we have printed nanowires as thin as 55 nm and have achieved a voxel generation rate of up to 1.7 × 10^9^ voxels/s. Thus, GP-TPL can be broadly applied to improve the rate and quality of nanoscale 3D printing and other projection-based fabrication techniques to manufacture functional devices for practical use.

## Methods

### Printer for grayscale projection two-photon lithography

Printing was performed on a custom-built system. The printer was driven by a Ti-Sapphire femtosecond NIR laser acquired from Spectra-Physics (Solstice Ace). The laser produces a linearly polarized beam with a center wavelength of 804 nm, FWHM spectral bandwidth of 41 nm, a pulse width of 35 fs, and a repetition rate of 5 kHz. The optical power of the beam was tuned with a combination of beam splitters and neutral density filters. The Gaussian intensity profile of the beam was transformed into a near-flat shape with a refractive beam shaper (πShaper). The DLP® LightCrafter^TM^ 6500 evaluation module, which was acquired from Texas Instruments, was used to implement the DMD-based digital mask. The DMD consists of a 2D array of individually switchable 1920×1080 square micromirrors that are each 7.6 μm wide. The DMD supports a maximum image refresh rate of 9523 Hz, which is higher than the repetition rate of the laser (i.e., 5 kHz). A commercially available tube lens with focal length of 200 mm was used as the collimating lens. For data shown in Figs. 1–6, an oil immersion objective lens (Olympus RMS60X-PFOD) with 60× magnification and 1.25 numerical aperture (NA) was used to focus the beam into the photoresist material. The objective lens had an optical efficiency of 0.73. A stacked three-axis X-Y-Z motion stage system (acquired from Physik Instrumente) was used to focus and position the substrate with respect to the objective lens. An Arduino board was used to synchronize the motion stages, the shutter for the laser beam, and the DMD for both continuous axial-scanning printing and layer-by-layer printing.

### Printing technique, post processing, and imaging of structures

For 3D printing, a droplet of the photopolymer was placed on top of a glass substrate that was coated with a thin film of indium-tin-oxide (ITO). The substrate was positioned on the X-Y-Z motion stage and moved in 3D space while the objective lens was held stationary. Printing was performed in the dip-in mode wherein the tip of the objective lens was submerged into the photopolymer resist. A series of projection images were transferred onto the DMD controller board, and the images were then projected in sequence in synchronization with the signals from the Arduino. During layer-by-layer printing, the Z-axis stage was moved in a step-by-step manner, and a new layer of the photopolymer was exposed after each Z-axis step. During printing via continual axial scanning, the Z-axis stage was moved continuously, without stopping for each layer, while the images were continually projected. The exposure times used for printing of various structures demonstrated in this study are available in the Supplementary Table S5.

The power of the beam was held constant at 177 nW per pixel for step-by-step printing (Figs. 3b, d and 4a, b) and 216 nW per pixel for all panels in Fig. 5. Experiments with the Gaussian beam profile (Fig. 4c) used non-uniform power per pixel values across the projection area due to non-uniform light intensity distribution. The power was measured immediately before the objective lens by projecting a white image. The power level was determined by the maximum power that could be incident on the DMD before it shut off due to overheating. For experiments with the Gaussian beam profile, the beam power was reduced by 18% to account for the increased transmission from the removal of the beam shaper, and the increased intensity at the center of the beam. Power used for printing the various structures for this study are listed in the Supplementary Table S5.

After exposure of all images, the substrate was transferred into a bath of propylene glycol methyl ether acetate (PGMEA) for 10 min. The substrate was then transferred into a bath of isopropyl alcohol (IPA) for another 10 min. These solvent baths dissolve the uncured liquid photopolymer and leave behind the cured material on the substrate. The substrate was then transferred into a bath containing a solution of 5% 2,2-dimethoxy-2-phenylacetophenone (Irgacure 651) in IPA. While submerged in this bath, the substrate was exposed to 365 nm UV light from a lamp for a duration of 20 min. The UV lamp produced a beam with an optical power of 4 W, and it was placed approximately 1 cm away from the printed structures. This photochemical UV curing step reduces mechanical failures, such as shrinkage and structural collapse, by improving the mechanical properties of the printed structures^60–62^. Mechanical failure generally results from the capillary forces that are generated during the solvent drying process. To minimize mechanical failure, it was ensured that the substrate was promptly transferred from one solvent bath to the next to prevent the structures from drying in air before photochemical curing. After UV exposure, the substrate was rinsed with clean IPA and then slowly dried inside a chamber with minimal airflow to reduce the capillary forces. For scanning electron microscopy (SEM) imaging, the printed structures were coated with a thin layer of gold (<10 nm thick) using a sputter coater. The Hitachi SU8230 SEM was used to image the printed 3D structures at 3 keV accelerating voltage.

### Photopolymer material

Custom photopolymer resists were prepared by mixing polyfunctional acrylate monomers, a custom photoinitiator, and quenchers. The monomer mixture contained (i) a mixture of pentaerythritol tetraacrylate, pentaerythritol triacrylate (PETA), and trimethylolpropane triacrylate that had a refractive index of 1.483, and (ii) bisphenol A ethoxylate diacrylate (BPADA) with average Mn∼468 – EO/phenol 1.5 that had a refractive index of 1.545. Both monomers were procured from Sigma Aldrich. These two monomers were mixed in a 35:65 ratio by weight to generate a monomer blend with a refractive index of 1.523, which is close to the refractive index of the oil immersion medium for the objective lens. This ratio minimizes spherical aberrations that could result from the refractive index mismatch between the resist and the immersion medium of the objective lens. The custom photoinitiator 4,4′-((1E,1′E)-(2-((2-Ethylhexyl)oxy)-5-methoxy-1,4-phenylene)bis(ethene-2,1-diyl))bis(N,N-dibutylaniline) was procured from a commercial custom chemical synthesis service provider. It was synthesized from precursors based on known literature procedures and it is expected to have a high two-photon absorption cross-section^63^. The concentration of the photoinitiator was either 0.25% (for data in Figs. 1, 3, 4 and 5a–c) or 1.0% (for data in Fig. 5d, e) by weight. For some experiments, the quencher 2,2,6,6-Tetramethylpiperidine 1-oxyl (TEMPO) was added at 500 parts per million (ppm) (Fig. 5c) or 1000 ppm (Fig. 5a, b) by weight.

### Empirical characterization of motifs

The FWHM size and the intensity of the motif patterns were empirically characterized by projecting a uniform array of motifs onto a reflective gold-coated glass substrate. The array consisted of 5 rows and 5 columns of identical motifs at a period of 70 pixels. The reflected NIR light was captured by the same objective lens that was used to project the image. Using a tube lens, the image of the gold surface was captured on a CMOS camera (DCC 3260C, Thorlabs). The protective IR filter on the camera was removed to ensure that the NIR light reflected from the gold film could be captured by the camera. During motif characterization, the power of the fs laser beam was reduced to such an extent that ablation of the gold surface was absent even over long exposures (~1 h). Additionally, the intensity was sufficiently low to ensure that appreciable amounts of optical signals could not be generated from two-photon photoluminescence of the gold film. This was verified by measuring the spectrum of the reflected light using a spectrometer (CCS200, Thorlabs). The intensity profile of each focal spot was computationally evaluated from the grayscale brightness of the optical image of the projected motif pattern. The FWHM diameter of the focal spot was evaluated from the intensity profile of the spot.

### Simulation of motif intensity profiles

The light intensity distribution on the focal plane, corresponding to each motif pattern, was computed through Fourier optics techniques that were implemented using the MATLAB software package. These techniques were applied to simulate the propagation of a single broadband femtosecond pulse through the optical projection system. The simulated system comprises the DMD, the collimating lens, and the objective lens. The instantaneous intensity at the focal plane was simulated by first separately evaluating the electric field for each wavelength using monochromatic coherent optical models and then summing up the contribution of each wavelength. The spatial intensity profiles of the motifs were computed by evaluating the time-averaged intensity at each spatial location by summing over the duration of the simulation (i.e., 4.3 ps). For each motif, the FWHM size was evaluated from the intensity profile and the peak intensity was evaluated from the highest intensity within the profile. In the plane of projection, the spatial grid for computation was discretized into units equal to the width of each DMD pixel. To simulate the intensity of each motif pattern, it was embedded at the center of a 128 × 128 pixel “off” image. An image larger than the motif was simulated to ensure that the entire focal spot was captured within the boundary of the simulation. The mathematical framework for the optical modeling is described in detail in our previous work^26^ and the codes are available elsewhere^64^. Specific model parameters for the simulations performed here are listed in the Supplementary Table S1. The specific motifs for the two objective lenses are listed in Supplementary Tables S2, S3.

## Supplementary information

Supplementary Information

Transparent Peer Review file

## Source data

Source data

## Data Availability

The data generated in this study are provided in the main text, Supplementary Information file and Supplementary Information/Source Data file. Any additional requests for information can be directed to, and will be fulfilled by, the corresponding author. Source data are provided with this paper.
