# Supplementary material for: Grayscale projection two-photon lithography using sub-diffraction motifs for ultrafast and precise nanoscale 3D printing
Source: Nat Commun. 2026 May 29;17:6981. doi: 10.1038/s41467-026-73782-x (PMC13392389; doi:10.1038/s41467-026-73782-x)
Supplement: Supplementary file 1 — Supplementary Information [file 41467_2026_73782_MOESM1_ESM.pdf]

Supplementary Information for

Grayscale projection two-photon lithography using sub-diffraction motifs for ultrafast and precise nanoscale 3D printing

Harnjoo Kim[1] and Sourabh K. Saha[1*]

[1]Scalable Technologies for Advanced Manufacturing Lab,
George W. Woodruff School of Mechanical Engineering,
Georgia Institute of Technology, Atlanta, GA 30332, USA

[*]Corresponding author email: ssaha8@gatech.edu

This PDF file includes:
Supplementary Text
Figures S1 – S26
Tables S1 – S5

S1. Optical parameters of the GP-TPL system for simulations

Table S1: Optical parameters of the GP-TPL system

**Parameter**	**Symbol**	**Numerical value**	**Units**
Repetition rate of laser	R_l	5	kHz
Central wavelength of laser	λ_0	804	nm
Spectral bandwidth of laser	$\Delta\lambda$	41	nm
Micro-mirror pitch on DMD	d	7.6	µm
Focal length of collimating lens	f_1	200	mm
Focal length of 60× objective lens	$f_{2,60}$	3	mm
Focal length of 40× objective lens	$f_{2,40}$	4.5	mm
Numerical aperture of 60× objective lens	NA_{60}	1.25	Non-dimensional
Numerical aperture of 40× objective lens	NA_{40}	1.3	Non-dimensional

S2. Determination of motif size

The diameter of the central Airy disk (D_d), i.e., the diffraction limit is given by:

$$D_d = \frac{1.22 \times \lambda_0}{NA_{60}} \tag{S1}$$

The magnification of the projection system is given by:

$$M_d = \frac{f_{2,60}}{f_1} \tag{S2}$$

Therefore, the pixel pitch in the projected image is given by:

$$d_p = d \times M_d \tag{S3}$$

Thus, from equations S1 and S3, the number of pixels (N) in the motif is given by:

$$N = \frac{D_d}{d_p} \tag{S4}$$

Substituting the numerical values of the parameters for the 60× lens, D_d = 785 nm, M_d = 0.015, d_p=114 nm, and N = 7 pixels.

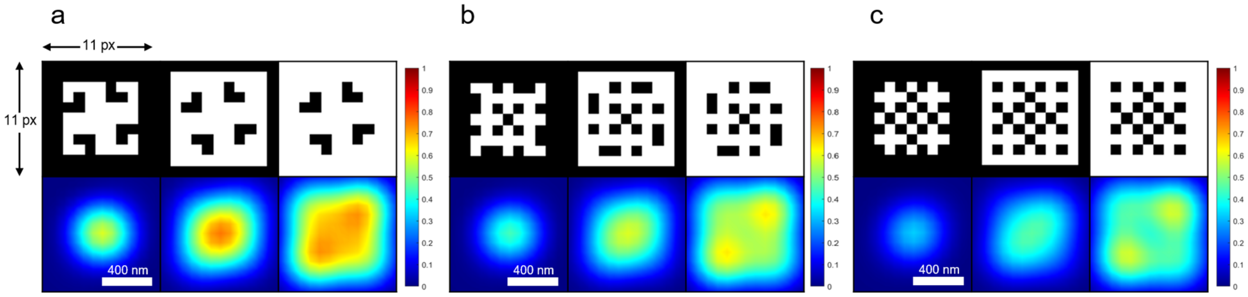

Figure S1: Effect of motif size on the intensity distribution for motifs that are wider than 7 pixels, for 60× objective lens. **a**, Projection of motif patterns equivalent to motif number 8, with motif sizes 7 × 7, 9 × 9, and 11 × 11. **b**, Projection of motif patterns equivalent to motif number 12, with motif sizes 7 × 7, 9×9, and 11×11. **c**, Projection of motif patterns equivalent to motif number 16, with motif sizes 7 × 7, 9×9, and 11×11.

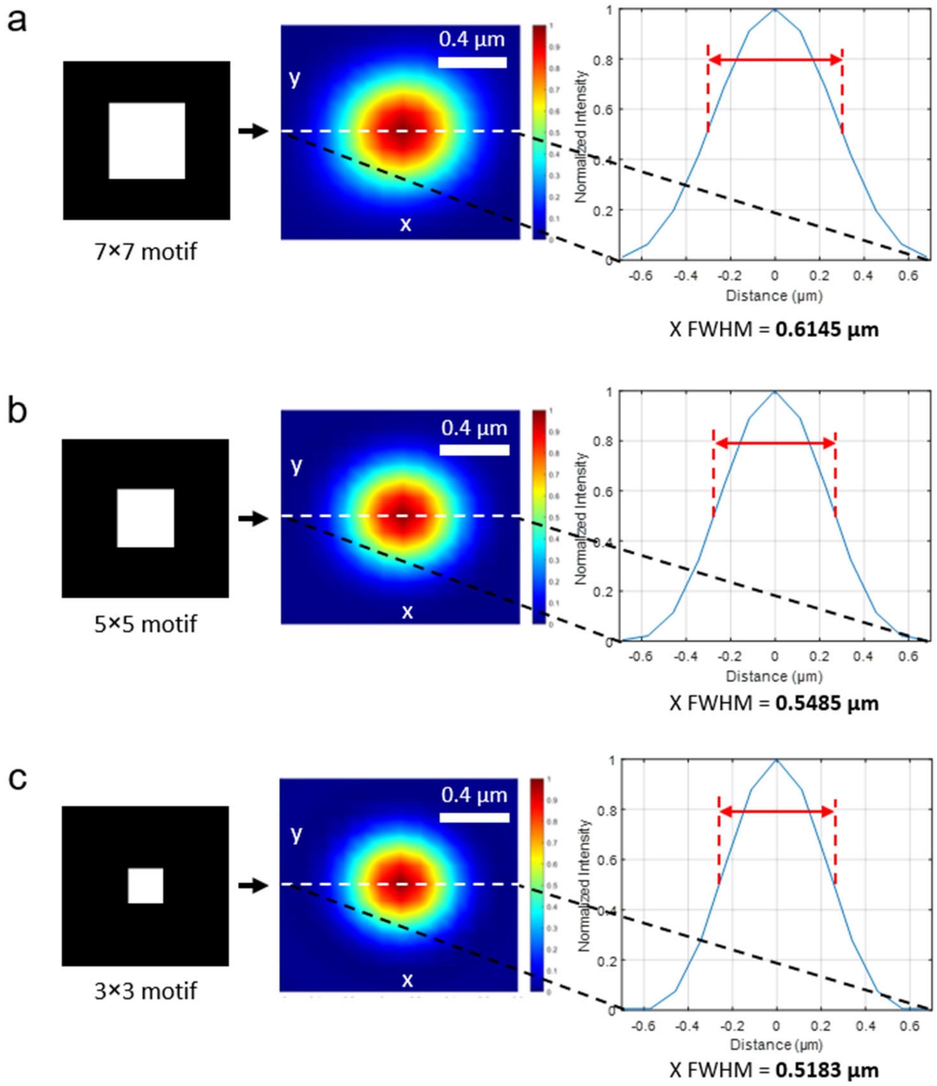

Figure S2: Effect of motif size on the intensity for a motif that is smaller than 7 × 7, for 60× objective lens. **a**, For a 7×7 fully bright motif. **b**, For a 5×5 fully bright motif. **c**, For a 3×3 fully bright motif. Peak intensities with 5×5 and 3×3 motifs are 53% and 12% of the peak intensity with the 7×7 motif, respectively.

S3. Determination of motif patterns

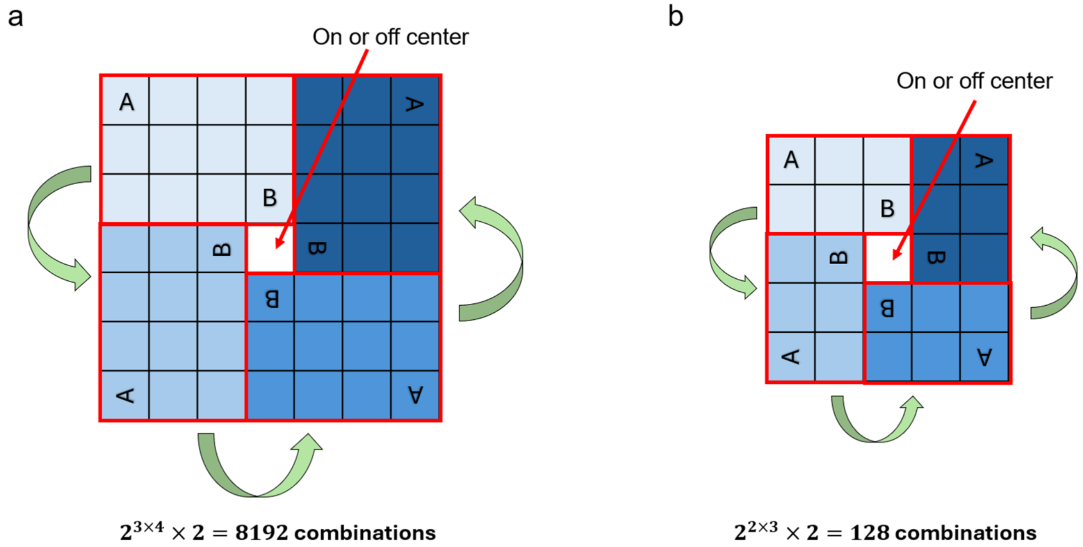

Figure S3: **a**, Schematic of 7 × 7 pixel group motif generation for use with 60× lens. **b**, Schematic of 5 × 5-pixel group motif generation for use with 40× lens.

The patterning of the 7 × 7 motifs is illustrated in Fig. S3 (a). Once all on/off combinations of 3 × 4 pixels in the sub-pattern are determined, the sub-pattern is rotated counterclockwise by 90 degrees to fill up the four 3 × 4-pixel corners of the 7 × 7 area. For all these combinations, the center pixel would be either turned on or off, resulting in a total of 13 bit (2^{13}) = 8192 unique motifs. These motifs are symmetric along two perpendicular axes going through the center point. Theoretically, there are a total of 2^{49} possible combinations of on/off mirrors within the 7 × 7 mirrors of the DMD. However, only a subset of these were selected using symmetry considerations. The same design concept is applied in patterning of the 5 × 5 motifs as shown in Fig S3(b).

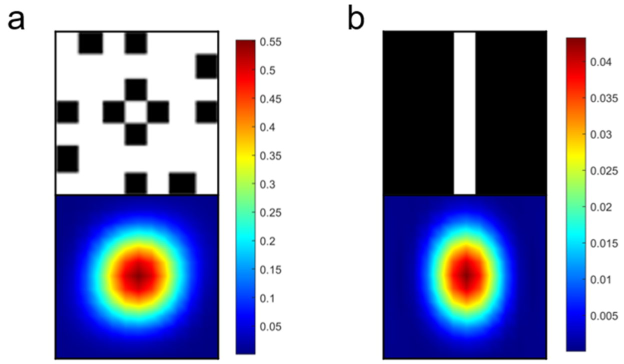

Figure S4: **a**, Symmetric motif (motif 9) and its intensity profile. **b**, Unsymmetrical motif and its intensity profile demonstrating lop-sided spot shape.

Fig S4 shows the effect of motif symmetry on the intensity profile. In Fig. S4(a), the spot is symmetrical as the full width half maximum (FWHM) size of the spot is 616 nm along the horizontal direction and 623 nm along the vertical direction. In contrast, the FWHM width of the spot in Fig. S4(b) is 491 nm along the horizontal direction and 662 nm in the vertical direction.

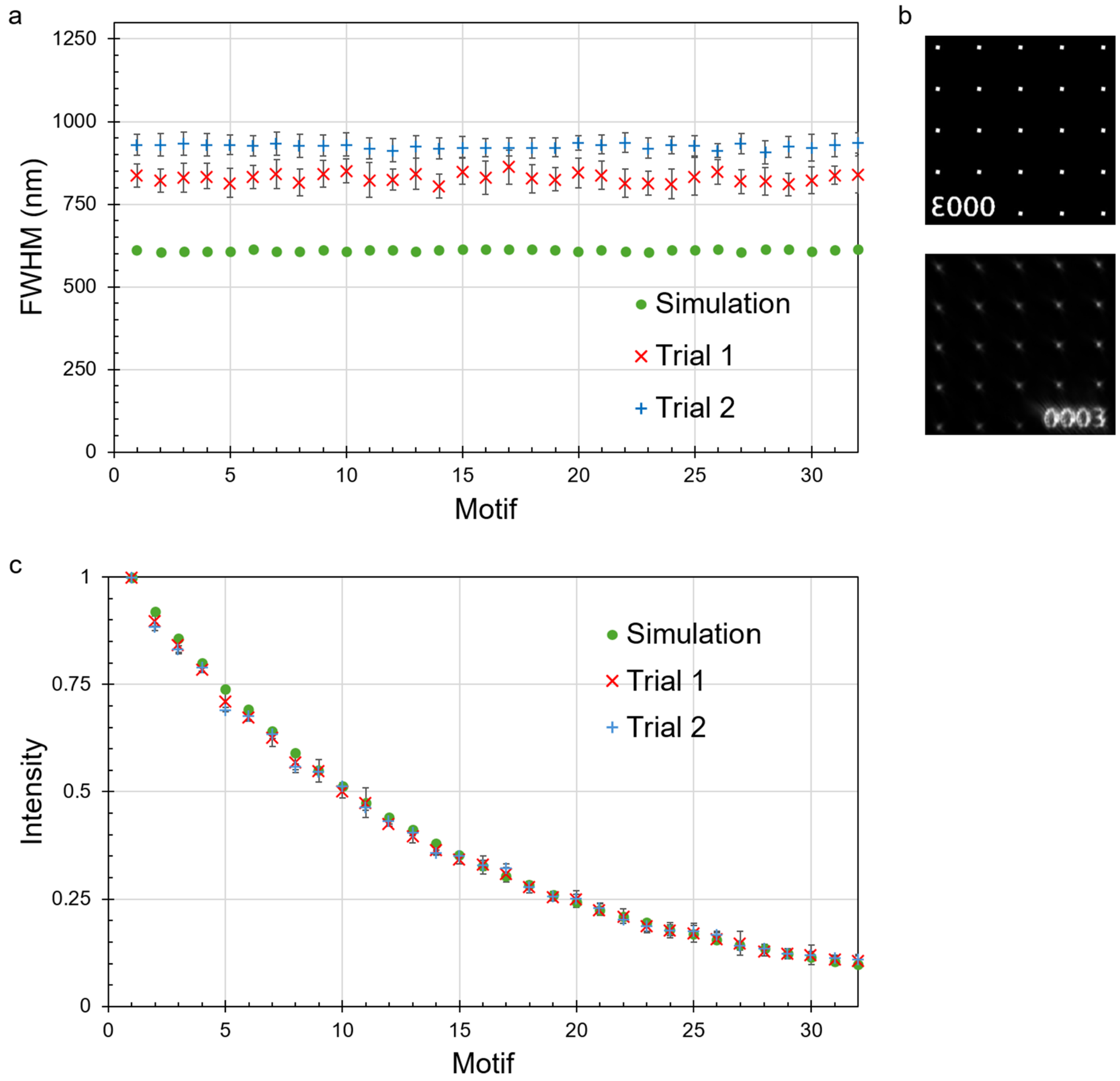

Figure S5: FWHM and peak intensity evaluation of 7 × 7-pixel group motifs from simulations and experiments. **a**, FWHM measurement versus motif number from simulation and experiment for 32 motifs of 7 × 7-pixel group. **b**, Top: Bitmap binary image of the grid patterns made with motif pattern #3. Bottom: Optical image of the projected pattern. **c**, Peak intensity measurement versus motif number from simulation and experiment for 32 motifs of 7 × 7-pixel group. Each experimental datapoint in **a** and **c** represents the mean from 5 measurements with error bar indicating 2 standard deviations.

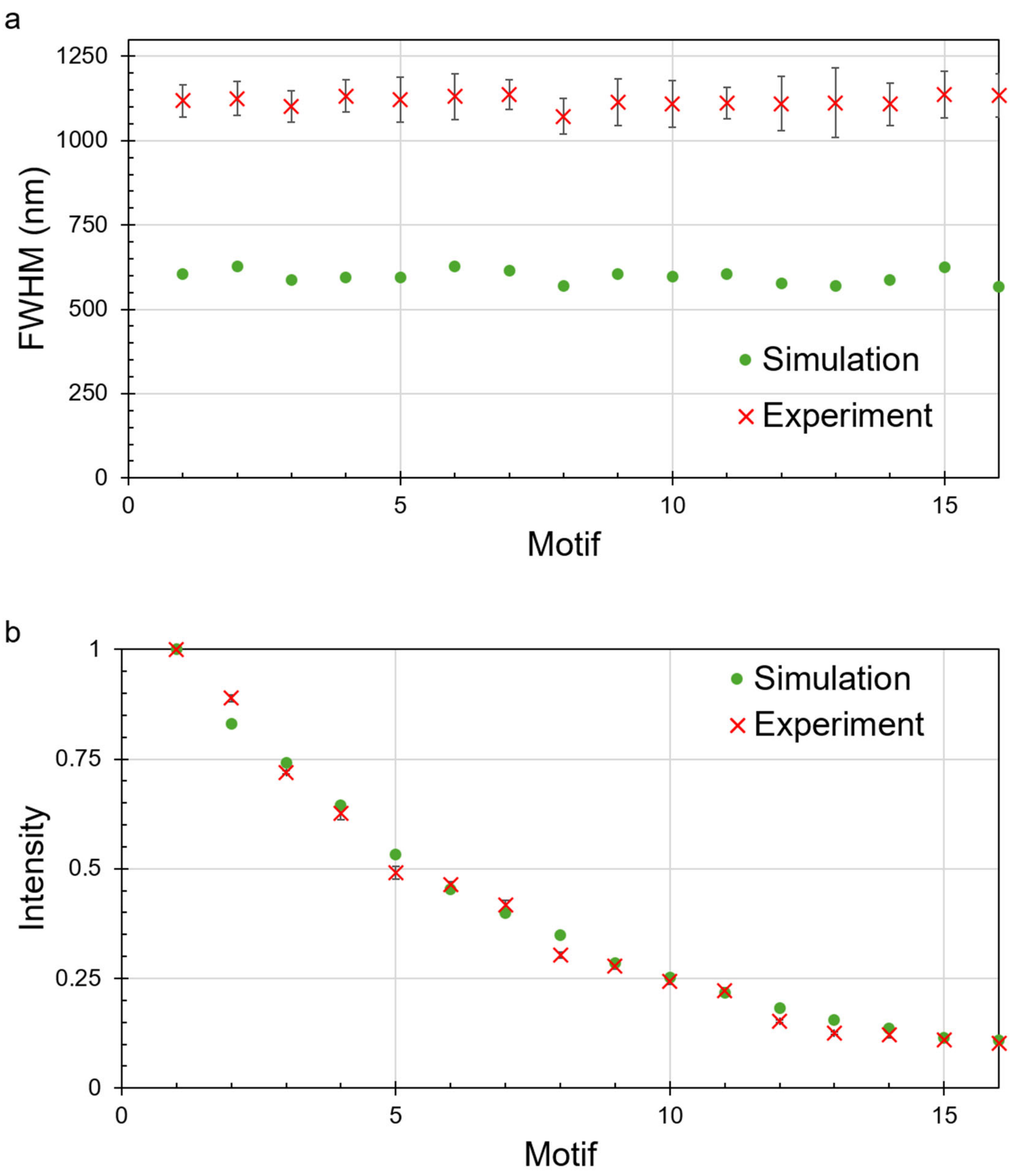

Figure S6: FWHM and peak intensity evaluation of 5 × 5-pixel group motifs from simulations and experiments. **a**, FWHM measurement versus motif number from simulation and experiment for 16 motifs of 5 × 5-pixel group. **b**, Peak intensity measurement versus motif number from simulation and experiment for 16 motifs of 5 × 5-pixel group. Each experimental datapoint in **a** and **b** represents the mean from 5 measurements with error bar indicating 2 standard deviations.

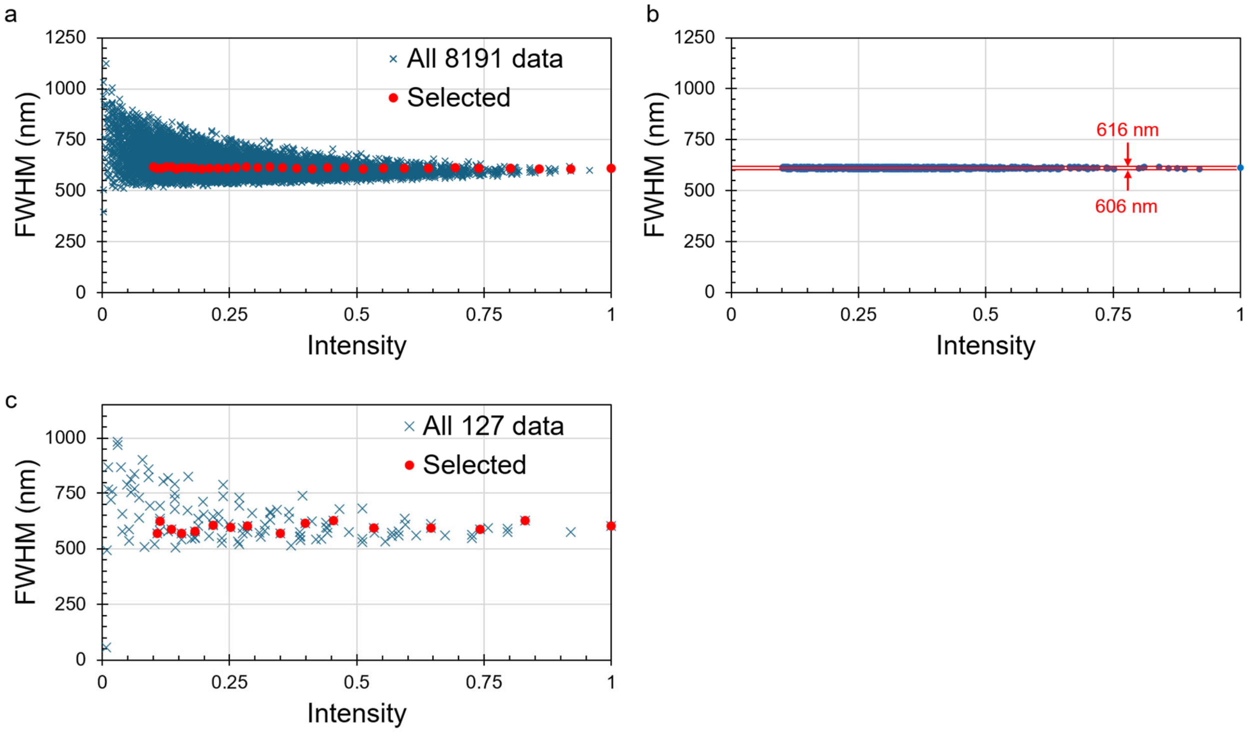

Figure S7: Simulated FWHM and intensity of all 2^{13} and 2^{7} sets for 60× and 40× lenses. **a**, Simulated FWHM and intensity of all 8191 motifs (excluding fully black pattern) for 60× lens. Selected 32 motif patterns of similar FWHM with 10 % to 100 % intensity range are indicated. Intensity is normalized by setting the intensity of the fully bright motif to 100% and the selected 32 motifs have their intensities spaced apart in equal percent steps of approximately 7.7 %. **b**, Extracted from plot **a**, but only showcasing selections of similar FWHM and intensity between 10% and 100%. 711 out of 8191 motifs remain in this range. **c**, Simulated FWHM and intensity of all 127 motifs (excluding fully black pattern) for 40× lens. Selected 16 motif patterns of similar FWHM with 10 % to 100 % intensity range are indicated. Intensity is normalized by setting the intensity of the fully bright motif to 100% and the selected 16 motifs have their intensities spaced apart in equal percent steps of approximately 16.6 %.

For ease of representation, each motif represented by the 7×7 or 5×5-pixel array has been converted into a hexadecimal number and listed in Tables S2 and S3. The square arrays were first flattened into a single sequence of 49 binary digits (for 7×7) or 25 binary digits (for 5×5), ordered row-by-row from top to bottom and left to right. Because each hexadecimal digit corresponds to 4 binary digits, the sequences were padded with three leading zeros to reach lengths of 52 and 28 bits for 7×7 and for 5×5, respectively, before conversion. Consequently, when converting the hexadecimal values back to binary, the first three padded zeros must be removed to recover the original 49 or 25-bit sequences.

Table S2: List of 5×5 motif patterns for 40× 1.3 NA objective lens

Motif number	Relative intensity (%)	Hexadecimal encoding
1	100.0	1FFFFFF
2	83.0	1FFEFFF
3	74.2	1BFBBFB
4	64.5	17F6DFD
5	53.3	0EAFEAE
6	45.4	0EDD76E
7	39.9	1BABABB
8	35.0	0AABAAA
9	28.5	0AD936A
10	25.2	0AAAAAA
11	21.7	1526C95
12	18.2	0859342
13	15.6	082AA82
14	13.6	0454544
15	11.4	0858342
16	10.8	1122891

Table S3: List of 7×7 motif patterns for 60× 1.25 NA objective lens

Motif number	Relative intensity (%)	Hexadecimal encoding
1	100.0	1FFFFFFFFFFFF
2	92.0	1F5FFFFFFFF5F
3	85.8	1FEEFFFFFEEFF
4	80.1	1DFFFF6DFFFF7
5	73.9	1E5FBFEFFBF4F
6	69.3	1EFBBFBBFBBEF
7	64.0	1BFDDEEEF77FB
8	59.2	1EEC9FFFF26EF
9	55.2	15FF7755DDFF5
10	51.3	1BEAFEAAFEAFB
11	47.5	0DAEF755DEEB6
12	44.2	1A5FAAEEABF4B
13	41.2	17E45DEF744FD
14	38.1	1E489FBBF224F
15	35.4	0BACD6D6D66BA
16	32.8	0ABBAAAAABBAA
17	30.5	13F94ABAA53F9
18	28.3	071375935D91C
19	26.2	0B06F4C65EC1A
20	24.4	09B3F4105F9B2
21	22.6	12EC16C6D06E9
22	21.0	070275935C81C
23	19.5	0A1588EE2350A
24	18.1	0B04C8EE2641A
25	16.8	0913F4005F912
26	15.6	08B3B4005B9A2
27	14.5	021B229289B08
28	13.5	0C11893923106
29	12.5	0A0494C65240A
30	11.6	0511492925114
31	10.7	00BD0244817A0
32	10.0	02B11482511A8

S4. Exemplary images of woodpiles printed with motifs

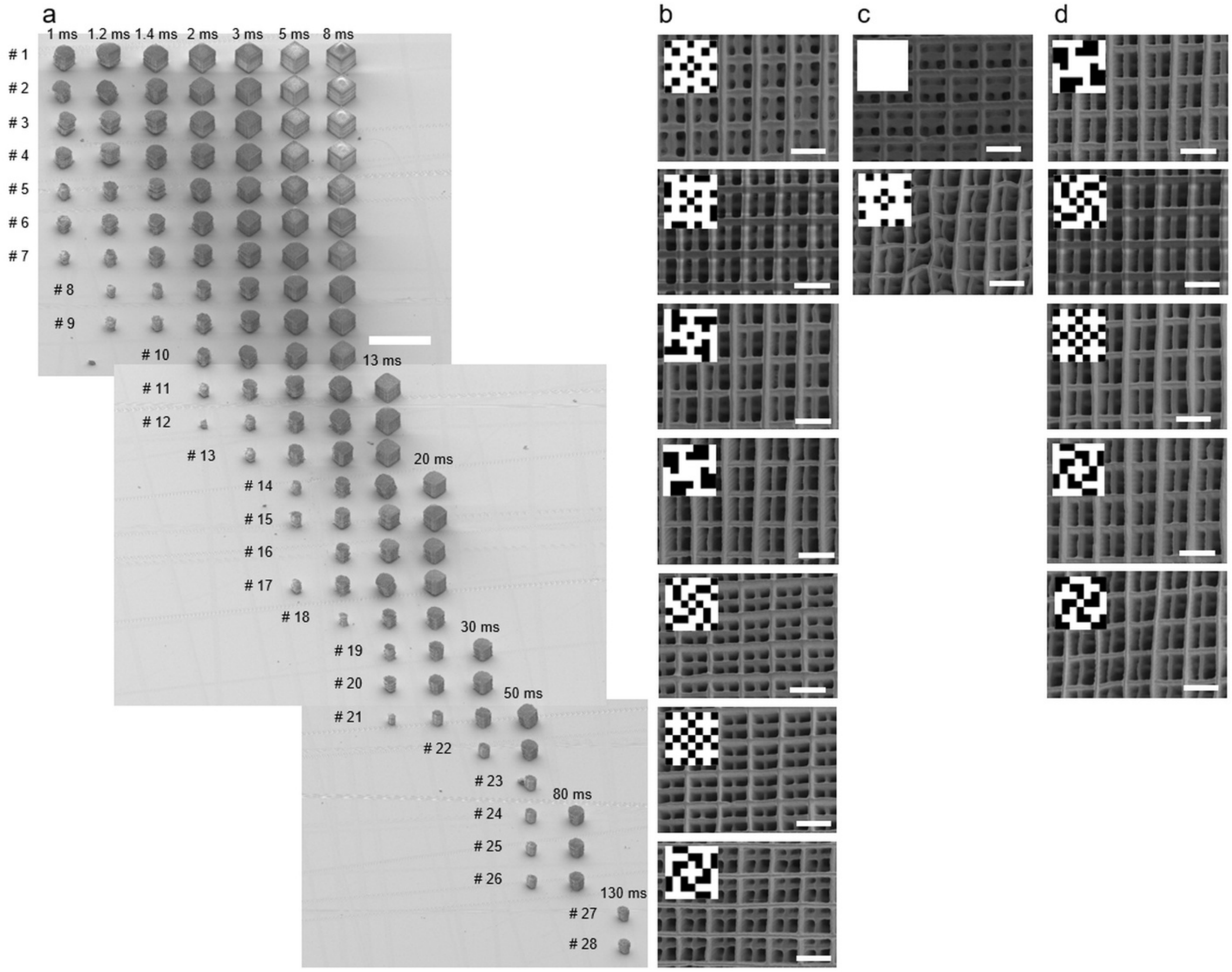

Figure S8: SEM images of woodpiles printed with various motifs, corresponding to Fig. 3b. **a**, Woodpile structures printed using a flat intensity profile laser at 177 nW/pixel. Each row corresponds to a different motif pattern used to substitute the grating lines (7-pixel line width), with motif #1 for the top row, motif #2 for the second row, and so on. Exposure time used for projections increases across columns from left to right: 1 ms, 1.2 ms, 1.4 ms, 2 ms, 3 ms, 5 ms, 8 ms, 13 ms, 20 ms, 30 ms, 50 ms, 80 ms, 130 ms. **b**, Close up view of the structure (top-down). Features are printed with a fixed exposure time of 13 ms using motifs #11 to #17 (top to bottom). **c**, Structures printed with a fixed exposure time of 2 ms using motif #1 (top) and motif #9 (bottom). **d**, Structures printed with a fixed exposure time of 20 ms using motifs #14 to #18 (top to bottom). Scalebar in **a**: 200 µm. Scalebars in **b**, **c**, **d**: 5 µm.

S5. Compensation of proximity effects in woodpiles with uniform sparsity

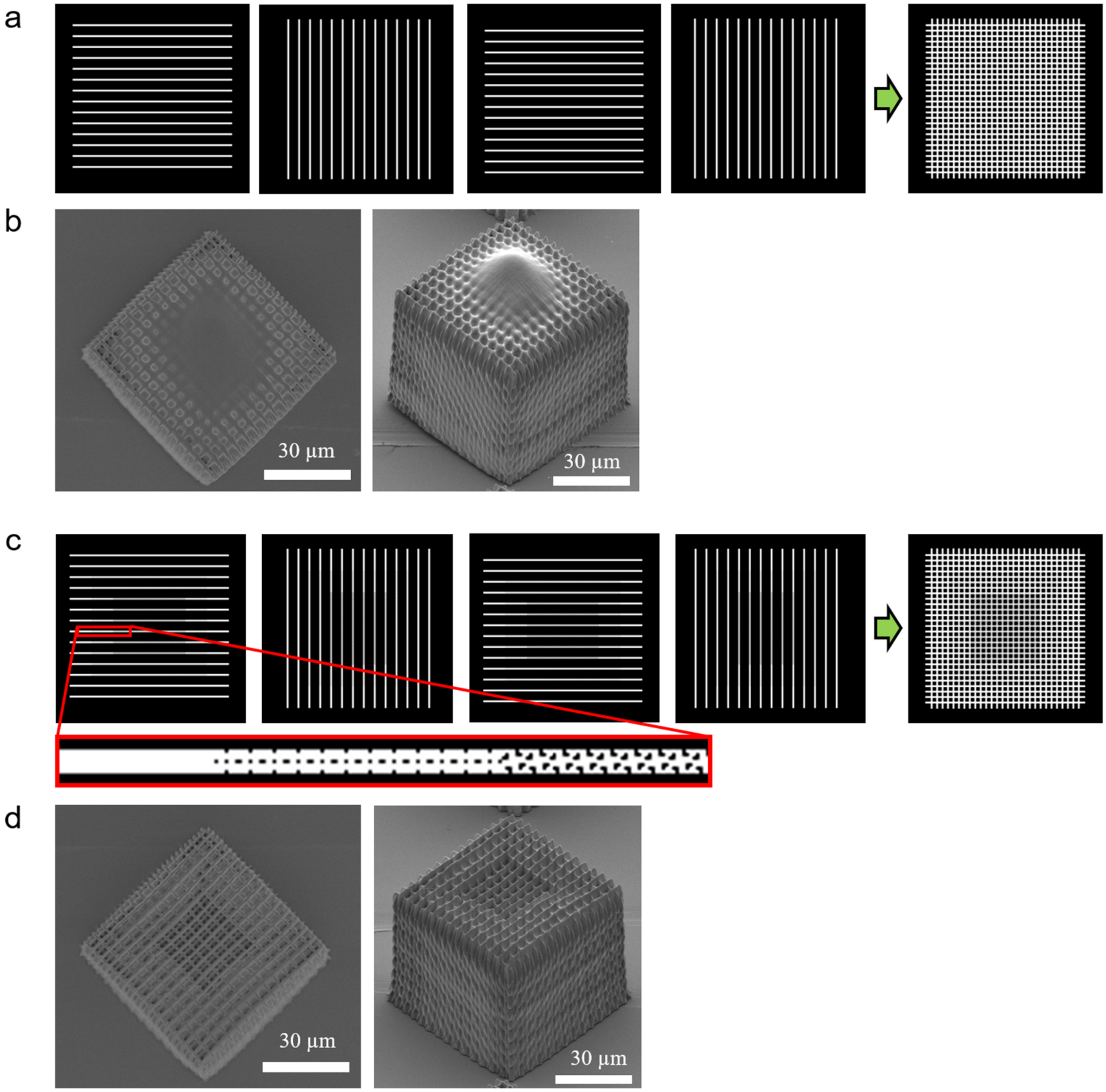

Figure S9: Compensation of over-polymerization in woodpiles with a single period. **a**, DMD images of full white grating lines. Combination of 4 patterns designed to be projected in sequence, one per layer, forms the pattern at the far right. **b**, Structure printed with full white lines shown in **a**. Top-down view and 45-degree tilted view. **c**, Grating lines substituted with three different grayscale motifs of 60%, 80%, and 100% intensity levels from center to the boundary. Combination of 4 patterns designed to be projected in sequence, one per layer, forms the pattern at the far right. **d**, Structure printed with lines substituted with grayscale motifs. Top-down view and 45-degree tilted view. 5 ms of exposure was used per layer for both prints in **b** and **d**.

S6. Compensation of proximity effects in woodpiles with non-uniform sparsity

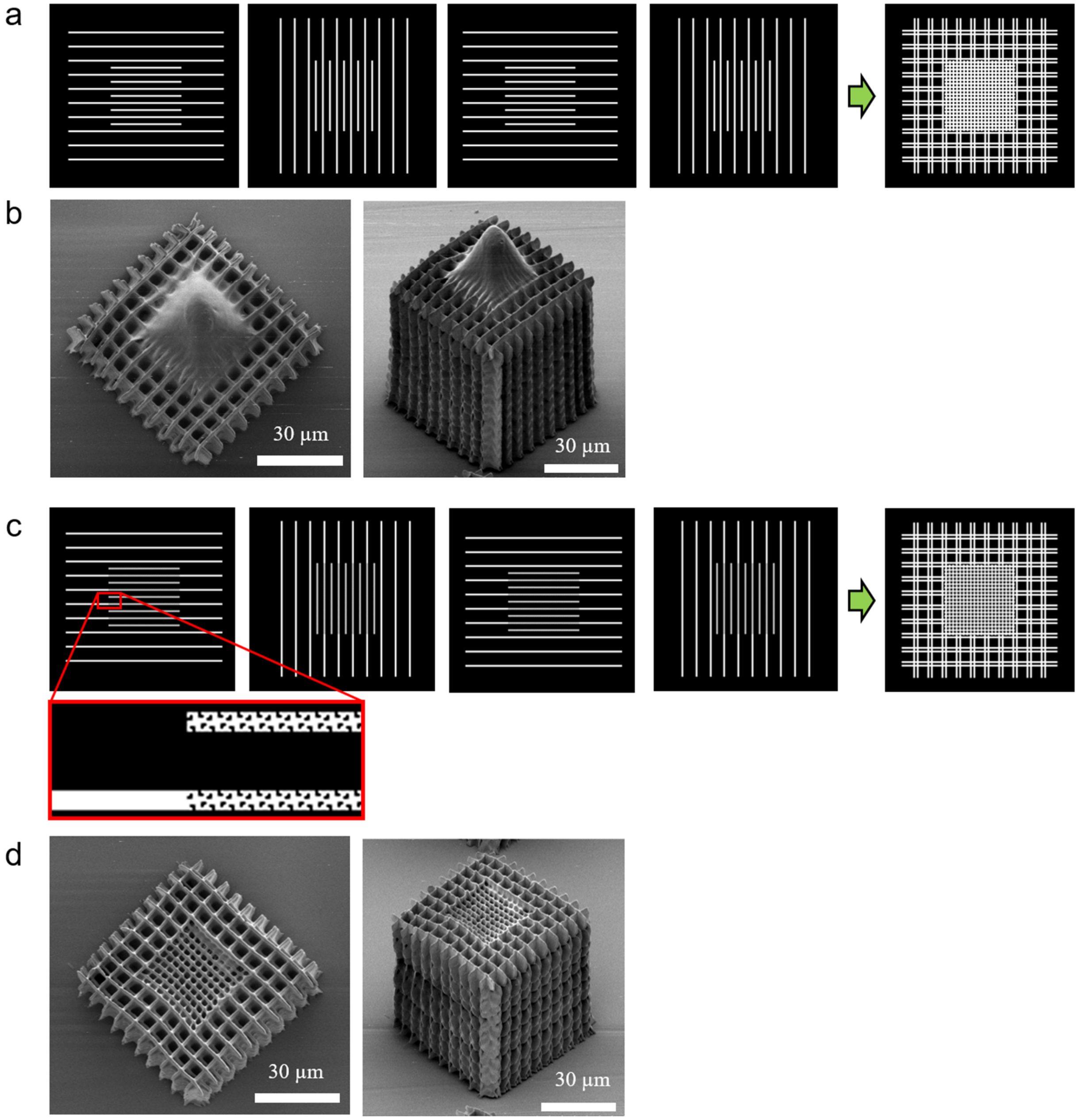

Figure S10: Compensation of over-polymerization in woodpiles with two different periods. **a**, DMD images of full white grating lines with twice the density in the central region. Combination of 4 patterns designed to be projected in sequence, one per layer, forms the pattern at the far right. **b**, Structure printed with full white lines shown in **a**. Top-down view and 45-degree tilted view. **c,** Grating lines of same shape as those in **a** but the lines in the central region are masked with grayscale motif of 60% intensity level. Combination of 4 patterns designed to be projected in sequence, one per layer, forms the pattern at the far right. **d**, Structure printed with lines substituted with grayscale motifs. Top-down view and 45-degree tilted view. 1.4 ms of exposure was used per layer for both prints in **b** and **d**.

S7. Compensation of over-printing in woodpiles with non-uniform illumination (60× objective)

The binary mask for compensation of non-uniform illumination was generated by capturing the intensity profile of the beam and then applying motifs to flatten the profile. Motifs of lower intensity level were applied in regions of higher intensity so that the projected intensity was uniform over the entire field. First, the intensity profile of the beam was captured using a beam profiler. As the size of the Gaussian beam produced by the laser was larger than the aperture of the beam profiler, the beam size was reduced using a telescopic beam reducer. This smaller beam and the central region of the original beam were imaged using the beam profiler. The intensity profiles were smoothed using Savitzky-Golay filtering and the FWHM size of the beams were measured. The telescope's magnification was inferred by equating the two FWHM measures. This magnification factor was applied to scale the intensity profile of the beam captured with the telescope to its original size. The beam was aligned so that the center of the beam was nominally at the center of the DMD. After the scale adjustments, only the area of the image that corresponds to the projection area in the DMD image is exported for further processing. We selected square projections of size 616×616 pixels, that were centered at the center of the DMD. This area is then segmented into smaller areas of 7×7 pixels. Over each 7×7-pixel group, the intensities (i.e., image brightness values) were averaged. The brightness values were normalized by setting the highest intensity to the value of 255. Our strategy to achieve uniform intensity distribution across the projection plane was to use the grayscale mask to reduce the intensity of the high-intensity regions to match the intensity of the lowest intensity region. The intensity of the motif (I_{motif}) corresponding to each 7×7-pixel area was obtained from the following relationship:

$$I_{motif} = \left(\frac{I_{7\times7}}{I_{min}}\right)^{-1} \tag{S5}$$

The intensity of each 7 × 7 pixel area ($I_{7\times7}$) is divided by the minimum intensity value within the 616 × 616 pixel projection area (I_{min}). Inverse of this value is the required intensity level of the motif. Out of the 32 previously selected motifs, the pattern with the closest peak intensity to this value is selected for substitution. The image obtained after the motif replacements over the entire area is the binary grayscale mask. Upon projection, this mask should generate uniform intensity across the projection area of 616 × 616 pixels. This was verified computationally through optical simulation and empirically through printing of woodpile structures. The horizontal and vertical grating line projection images required for printing of woodpile structures are generated using the same approach. Detailed steps of mask generation are illustrated in Fig. S12.

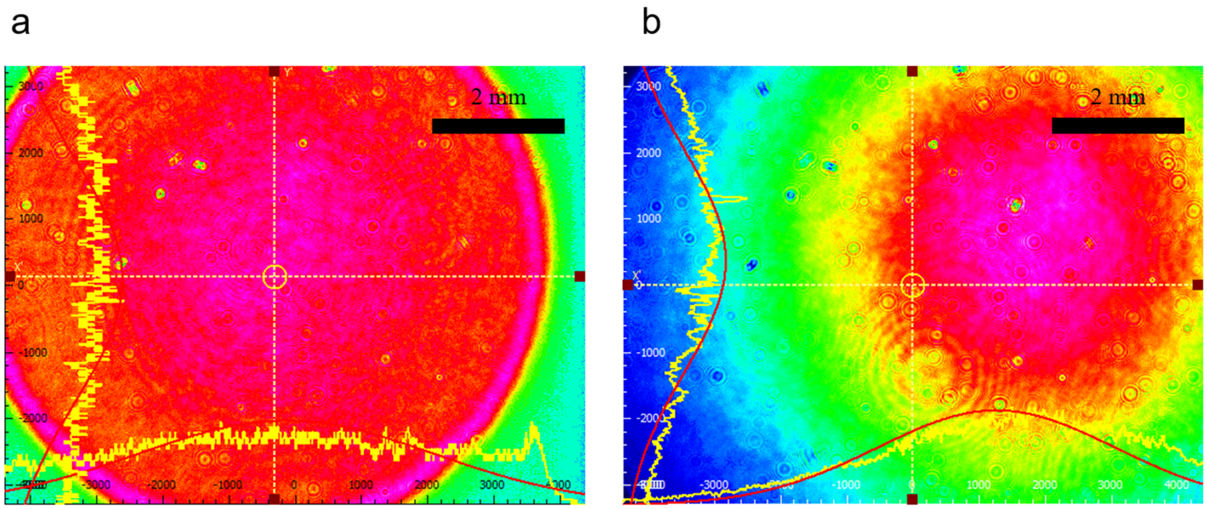

Figure S11: **a**, Near-flat intensity profile of the beam after the beam exits the beam shaper. **b**, Gaussian intensity profile of the femtosecond laser beam before it enters the beam shaper.

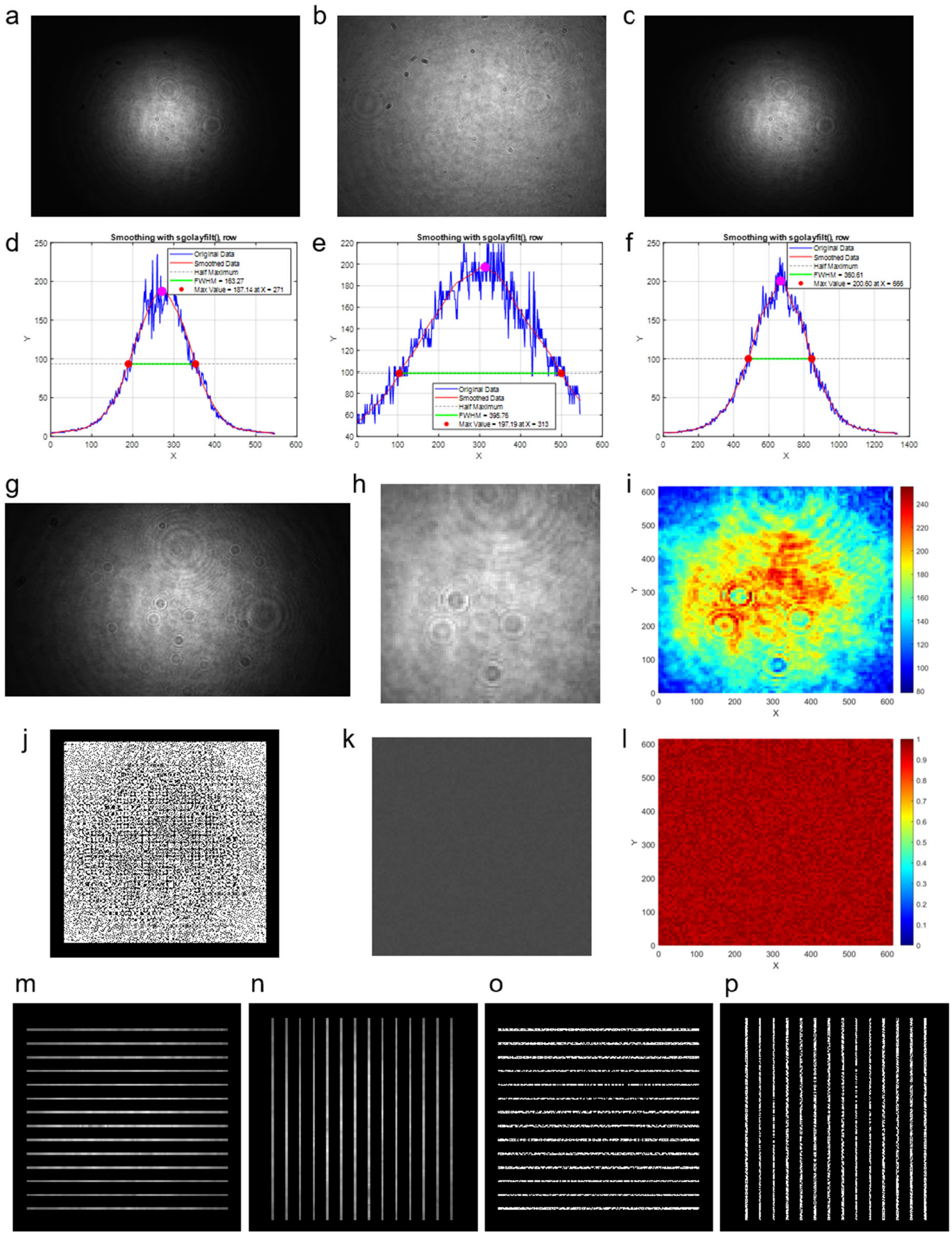

Figure S12: Details on grayscale mask generation for compensation of non-uniform illumination. **a**, Intensity profile of scaled-down Gaussian beam. **b**, Intensity profile of central region of Gaussian beam without beam reduction. **c**, Intensity profile of the entire Gaussian beam, scaled by the magnification of the beam reducer. **d**, FWHM of the scaled down Gaussian beam from **a**, measured after smoothing the noise

of the cross-section intensity profile using Savitzky-Golay filter. **e**, FWHM of the original Gaussian beam from **b**. **f**, FWHM of the scaled-up Gaussian beam from **c**. **g**, Gaussian profile re-sized & truncated to match the size of the DMD projection image profile (1920 × 1080 pixels). **h**, Cut out of the profile in 616 × 616 pixels, which matches the projected pixel area of the grating lines. **i**, Intensity distribution of the profile in **h**, represented in color. **j**, Mask generated with motif substitution in each 7 × 7 group pixels within the 616 × 616 pixel area. **k**, Expected intensity distribution profile of the 616 × 616-pixel area after each 7 × 7 group pixels is multiplied by the mask intensity value of the corresponding location. **l**, Expected intensity distribution of **k** represented with color. **m**, Image obtained by multiplying an image of horizontal grid lines with the measured intensity profile of the beam. This area is pixelated in groups of 7 × 7 pixels and normalized to the highest average intensity. **n**, Same as **m** but with vertical grating pattern. **o**, Mask for horizontal grating lines generated by substituting motifs on the lines shown in **m**. **p**, Same as **o** but mask generated for vertical lines.

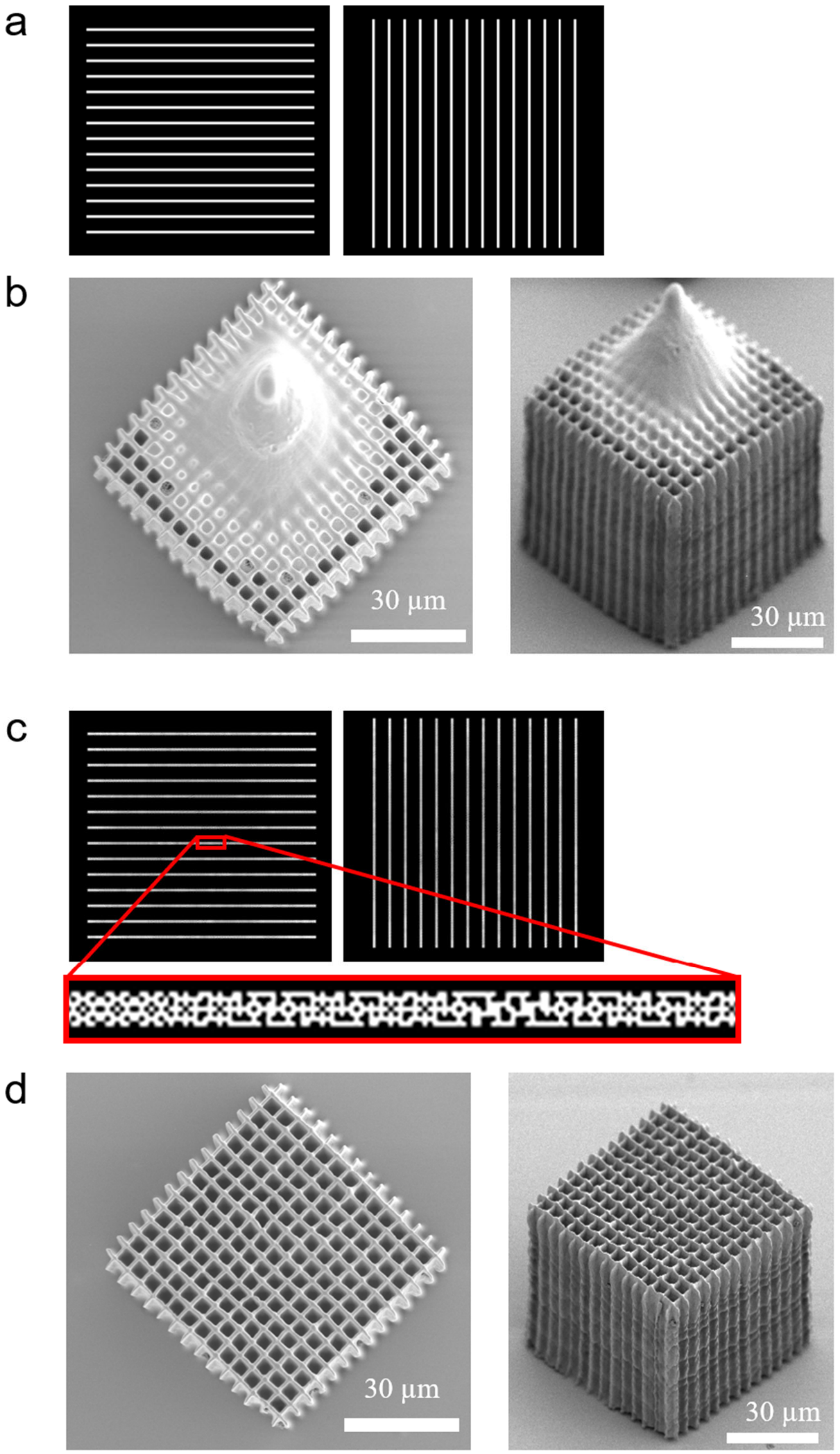

Figure S13: Compensation of over-polymerization due to non-uniform illumination originating from a Gaussian beam profile. **a**, DMD images of full white grating lines. **b**, Structure printed with full white lines shown in **a**. 2 ms of exposure was used per layer. Top-down view and 45-degree tilted view. **c**, Grating lines of same shape as those in **a**, but substituted with various grayscale masks chosen from the 32 motifs patterns from Fig. 2b. **d**, Structure printed with lines substituted with grayscale motifs. 3 ms of exposure was used per layer. Top-down view and 45-degree tilted view.

S8. Generalizability of GP-TPL

S8.1. Compensation for over-printing in woodpiles with non-uniform illumination (40× objective)

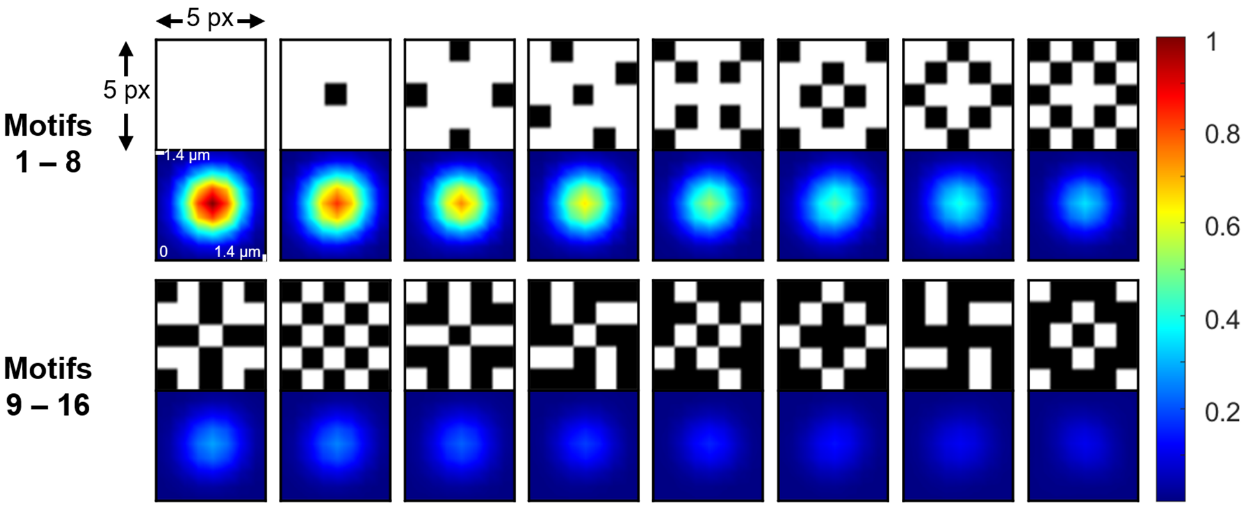

Figure S14: Full list of 16 motifs of 5 × 5-pixel group and simulated intensity profiles. Intensities are normalized to the peak intensity of the first motif.

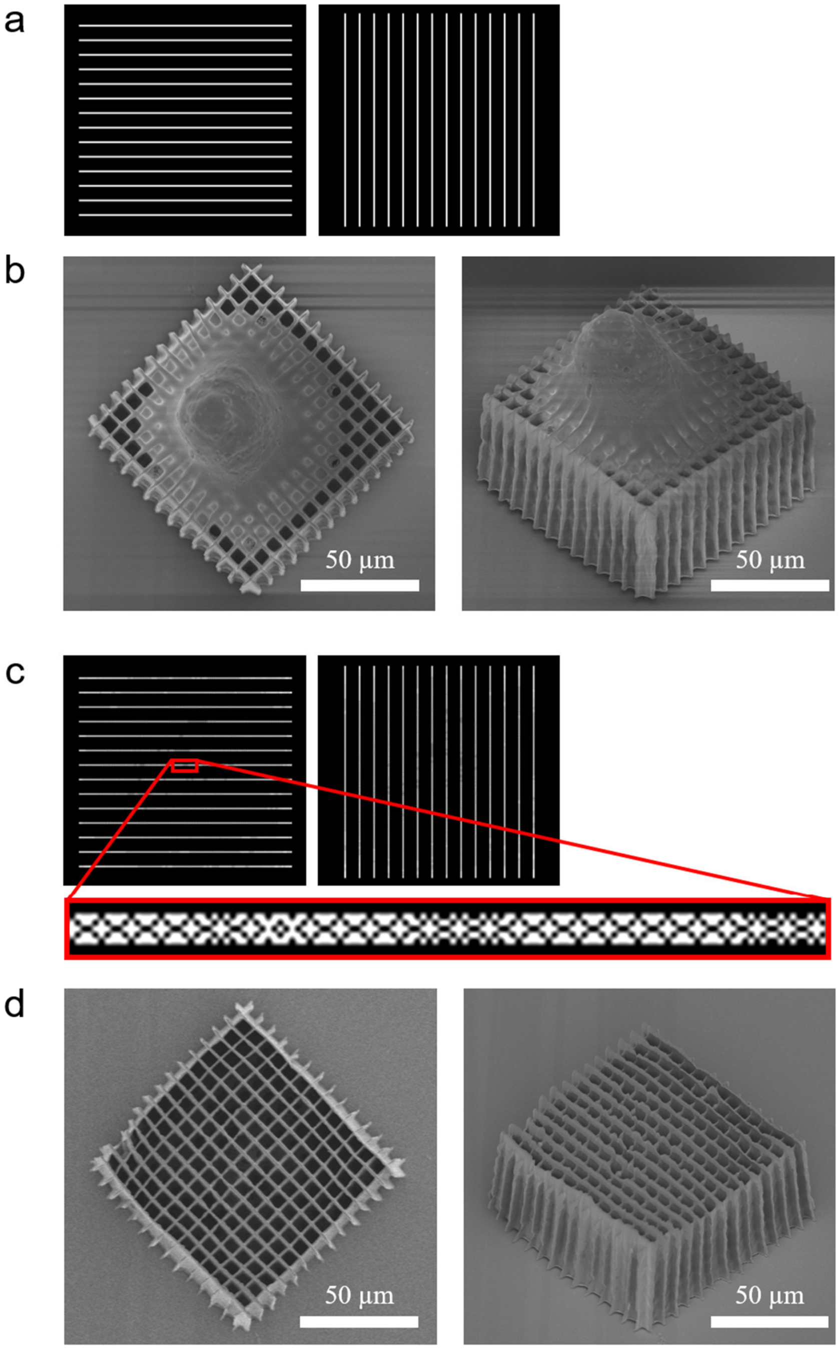

Figure S15: Compensation of over-polymerization due to non-uniform illumination originating from a Gaussian beam profile, using 40× objective lens. **a**, DMD images of full white grating lines. **b**, Structure printed with full white lines shown in **a**. 2 ms of exposure was used per layer. Top-down view and 45-degree tilted view. **c**, Grating lines of same shape as those in **a**, but substituted with various grayscale masks chosen from the 16 motifs patterns from Fig. S14. **d**, Structure printed with lines substituted with grayscale motifs. 8 ms of exposure was used per layer. Top-down view and 45-degree tilted view.

S8.2. Compensation for excessive metal ablation with non-uniform illumination (60× objective)

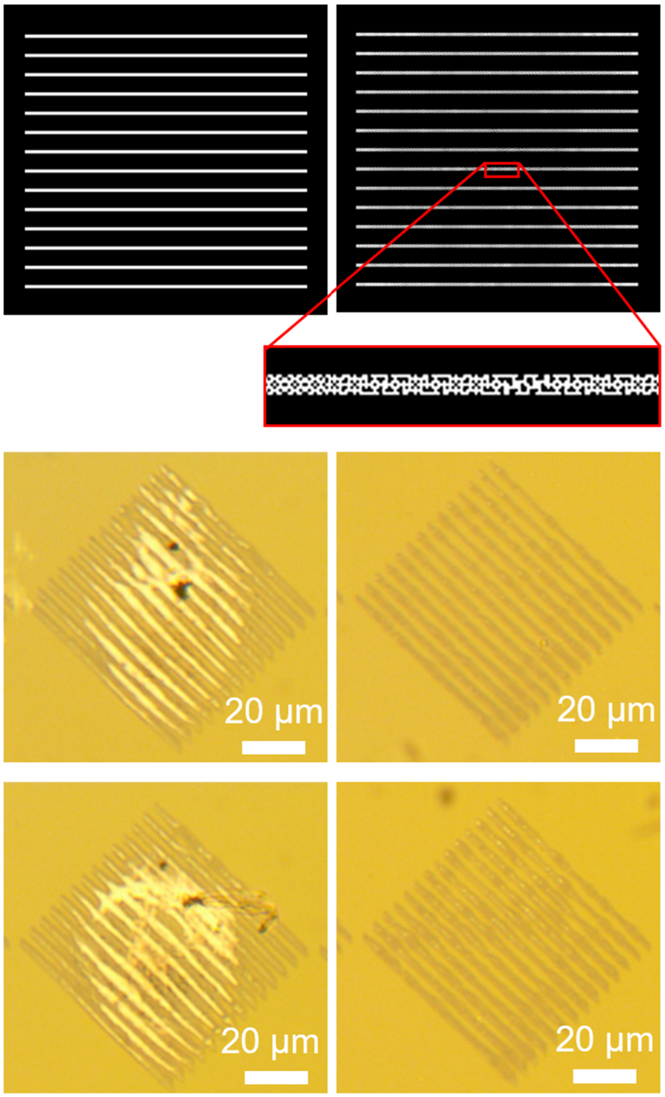

Figure S16: Compensation of excessive ablation of gold thin film due to non-uniform illumination arising from the Gaussian beam profile, using 60× objective lens. Grayscale mask contained the first 17 motifs of 7 × 7-pixel groups where intensities vary from 100% to 30%. Middle row shows ablated gold surfaces after 1.6 ms of exposure and bottom row shows ablated gold surfaces after 1.8 ms of exposures. Ablations on the left are the result of projecting 7-pixel width fully white grating lines and the ablations on the right are the result of projecting grayscale 7-pixel width grating lines comprising various motifs.

S8.3. Compensation for proximity effects observed with different resists

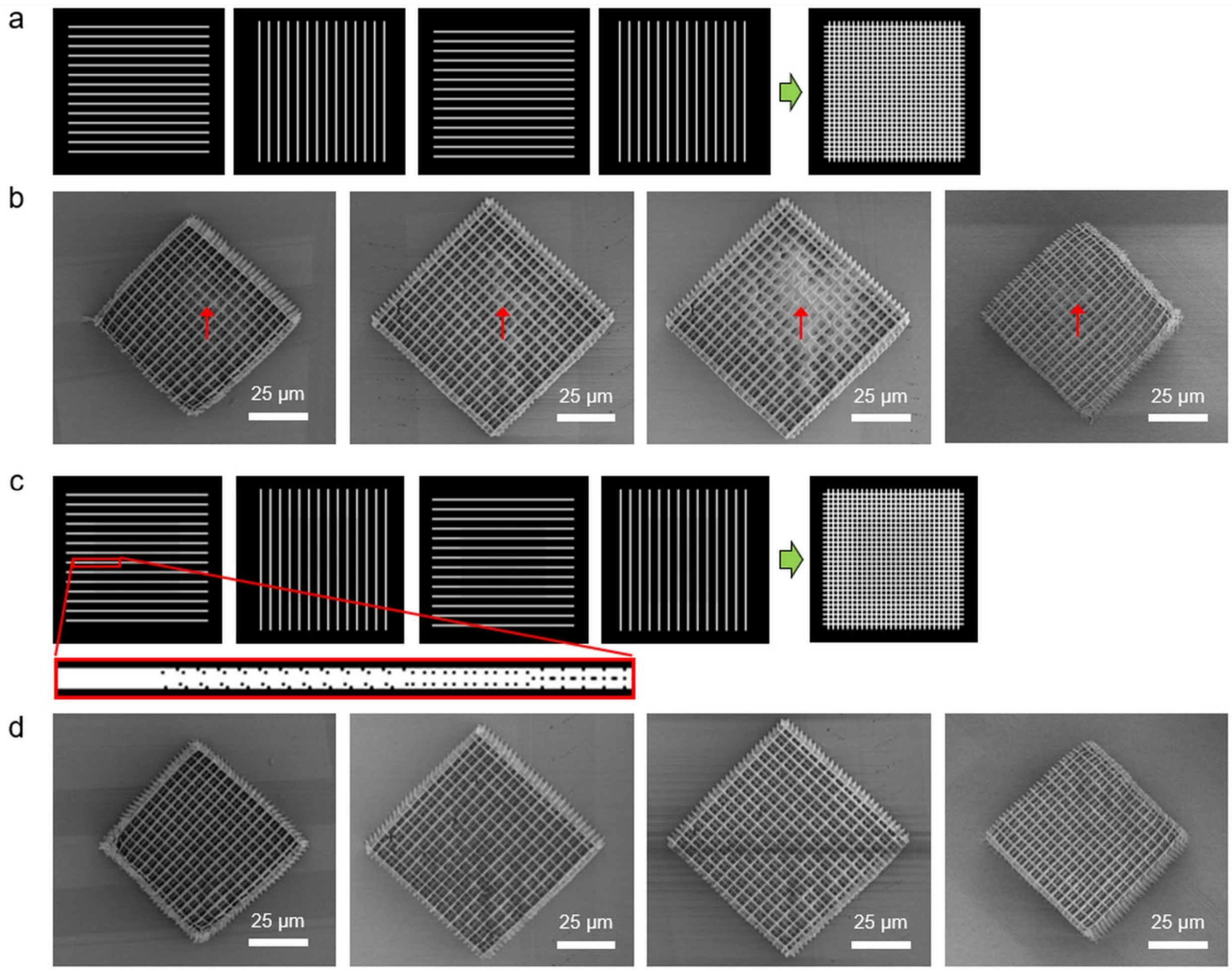

Figure S17: Compensation of over-polymerization in woodpiles using the same GP-TPL mask but with different resist compositions. **a**, DMD images of full white grating lines. Combination of 4 patterns designed to be projected in sequence, one per layer, forms the pattern at the far right. b, Top-down views of the structures printed with full white lines shown in a. Structure in the first image (left to right) was printed using a resist with 0.1% photoinitiator and 500 ppm 4-methoxyphenol (MEHQ) inhibitor and 3 ms exposure per layer. Structure in the second (or third) image were printed with 0.25% photoinitiator with 1000 ppm TEMPO inhibitor; 5 ms (or 8 ms) exposure per layer was used. Structure in the fourth image was printed with 0.25% photoinitiator without any additional inhibitor; 1.4 ms exposure per layer was used. **c**, Grating lines substituted with four different motifs of 80%, 86%, 92%, and 100% intensity levels from center to the boundary. Combination of 4 patterns designed to be projected in sequence, one per layer, forms the pattern at the far right. **d**, Top-down views of the structures printed with lines substituted with grayscale motifs as shown in **c**. Structures in the same column were printed with the same resist and exposure time (e.g., first image was printed using the resist containing 0.1% photoinitiator and 500ppm MEHQ inhibitor and 3 ms exposure per layer).

S9. Grayscale compensations to enable single-pulse-per-layer rapid continuous printing

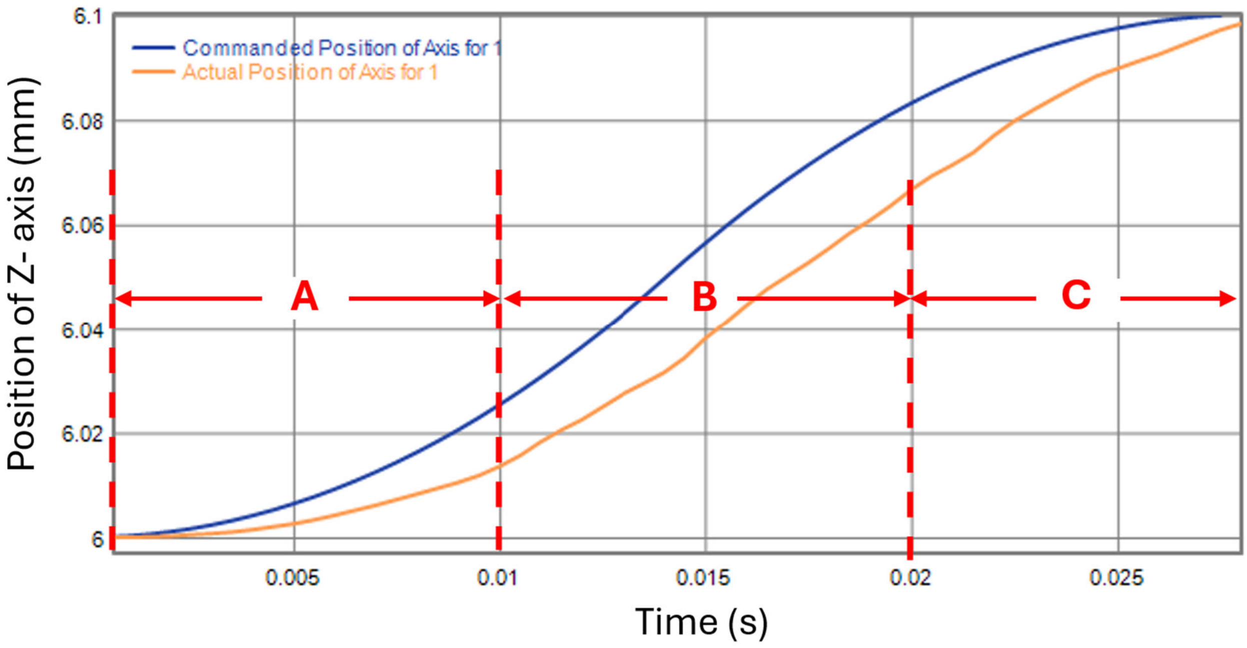

Figure S18: Position of the Z-axis (i.e., axial scanning axis) stage versus time, divided into three sections. Section A: Acceleration zone, section B: Constant speed zone, and section C: Deceleration zone.

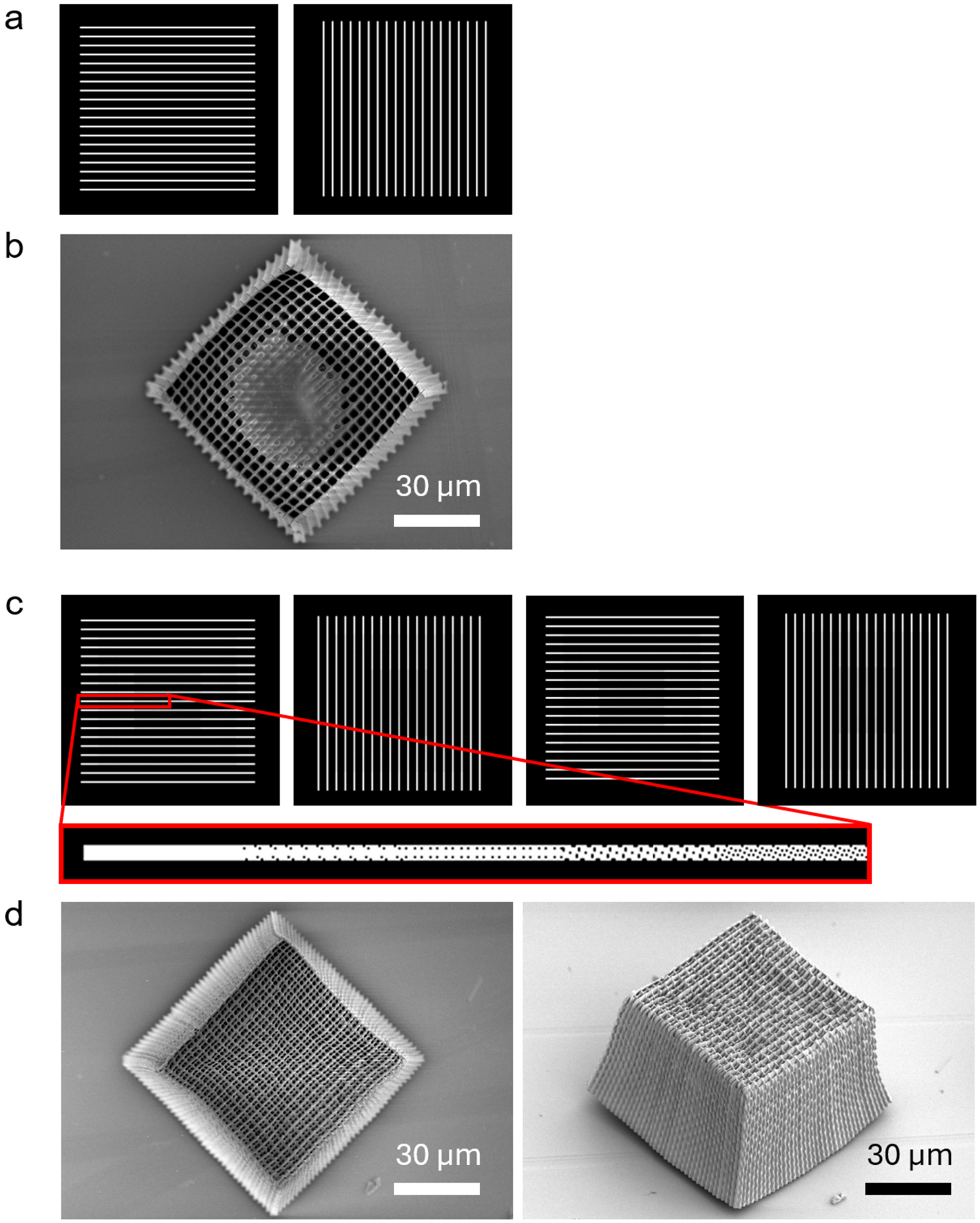

Figure S19: Demonstration of proximity effect correction via GP-TPL in continuous axial-scanning single-pulsed projection mode of printing. **a**, Projected bitmap images of 7-pixel wide full white horizontal and vertical lines. **b**, Top-down view of the resulting structure after alternating projections of bitmap images **a**. **c**, Bitmap images of 7-pixel wide lines substituted with motifs of 5 different intensity levels: 100%, 92%, 86%, 74%, 64%, ordered from the boundary to the center. **d**, Top-down view and 45-degree tilted view of the structure printed by projecting images from **c**.

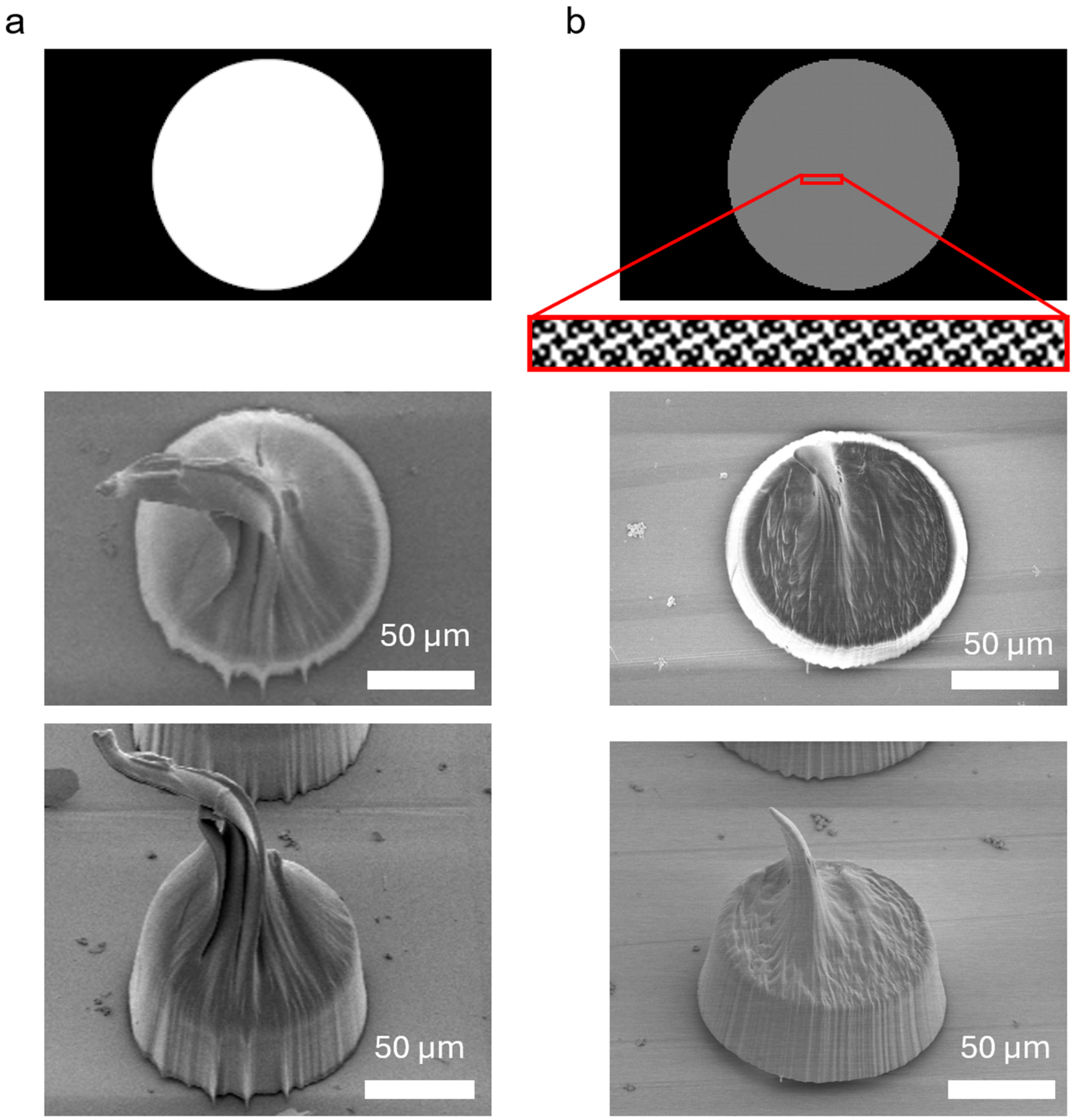

Figure S20: Continuous axial-scanning single-pulsed printing of fully dense cylinder structure. **a**, Top: full white projection image for printing solid cylinder. Middle: Top-down view of the solid cylinder printed with **a**. Bottom: 45-degree tilted view of the solid cylinder printed with **a**. **b**, Top: projection image for printing solid cylinder after substituting motif with 26.3% intensity representation throughout the feature area. Middle: Top-down view of the solid cylinder printed with **b**. Bottom: 45-degree tilted view of the solid cylinder printed with **b**.

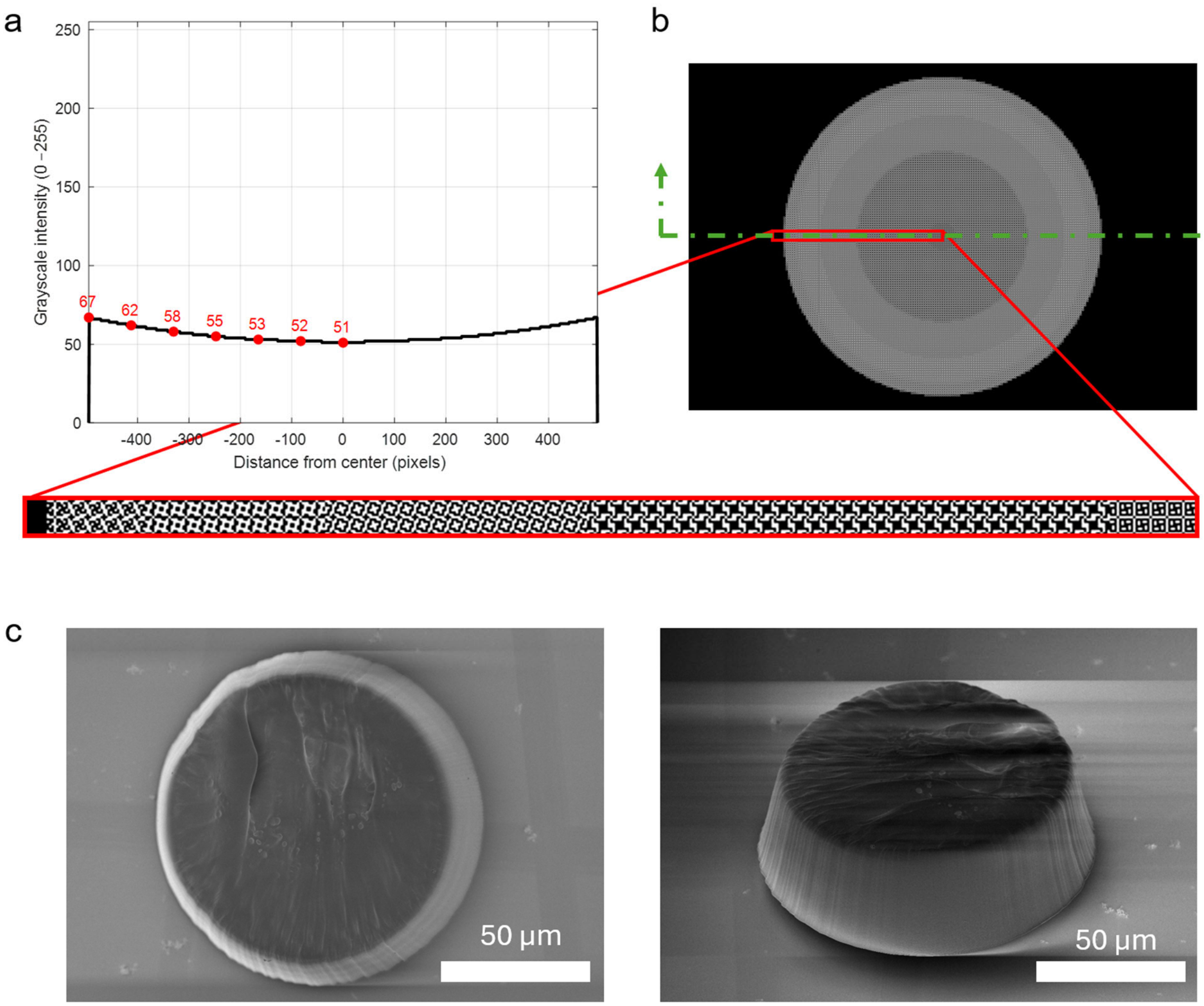

Figure S21: Continuous axial-scanning single-pulsed printing of solid cylinder using GP-TPL. **a**, Cross section of the desired intensity profile. **b**, Projection image of the solid cylinder after substituting various motifs. 5 different motifs ranging from 26.3% to 19.5% intensity levels were used from the edge of the cylinder to the center. Closeup inset image shows the motifs substituted across the radius of the cylinder. **c**, Left: top-down view of the solid cylinder printed using projection image shown in **b**. Right: 45-degrees tilted view of **c**.

S10. Comparison of physics-based simulations and approximations for prediction of motif levels

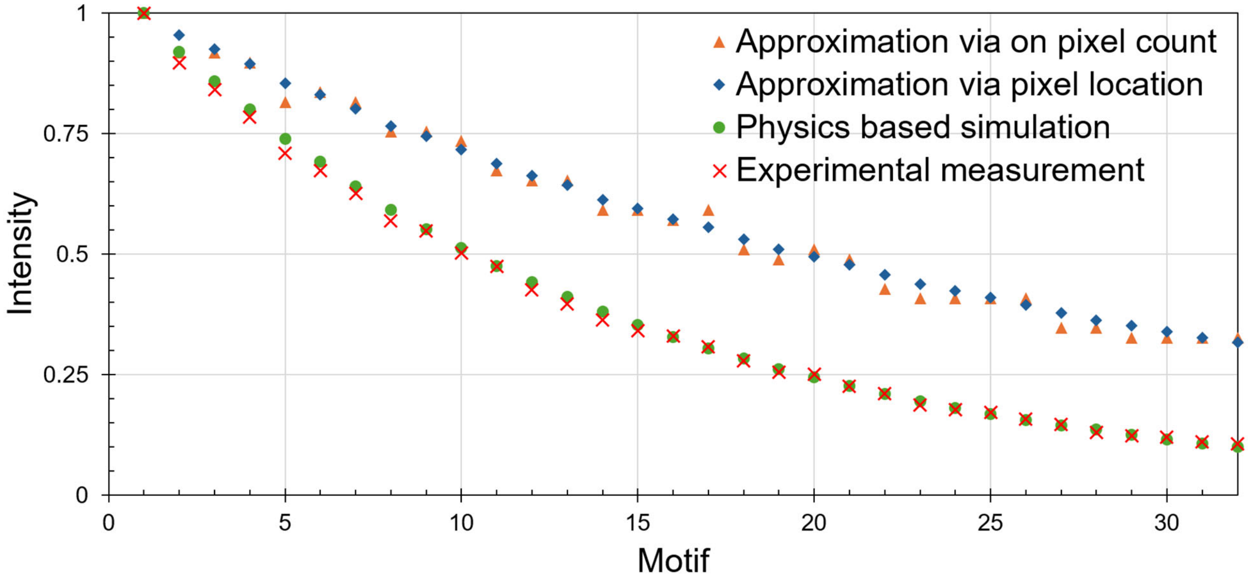

Figure S22: Comparison of measured and simulated intensity of motifs versus motif number for the 32 7×7-pixel motifs. Physics-based simulation refers to Fourier optics simulation of intensity. Approximation via on pixel count refers to summing up the number of on pixels to estimate the total power of the motif and approximation via pixel location refers to weighting the contribution of each on pixel by its location from the center using a Gaussian profile and then summing up the contributions to estimate the total power of the motif.

S11. Comparison of rate and resolution of two-photon lithography (TPL) techniques

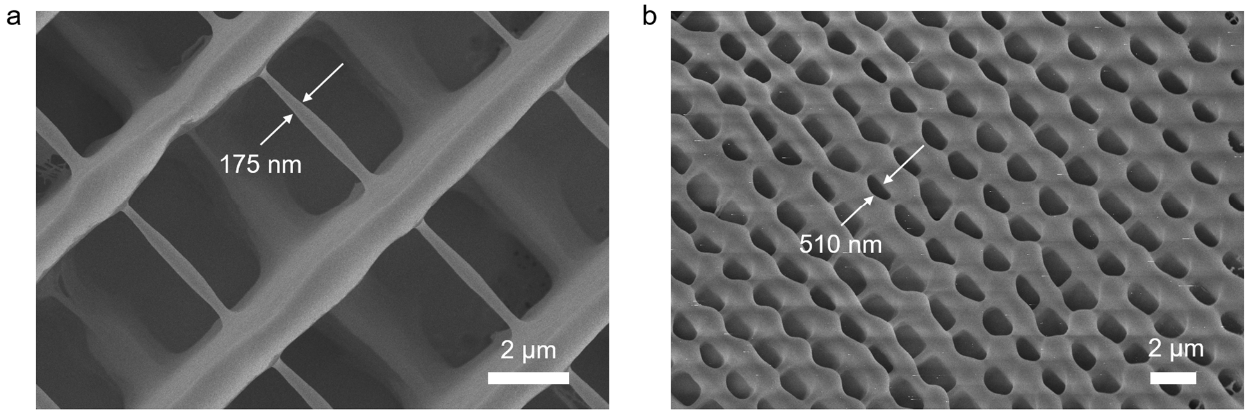

Figure S23: **a**, Demonstration of fine feature resolution through printing of suspended nanowires via GP-TPL. **b**, Demonstration of fine porosity during printing with GP-TPL.

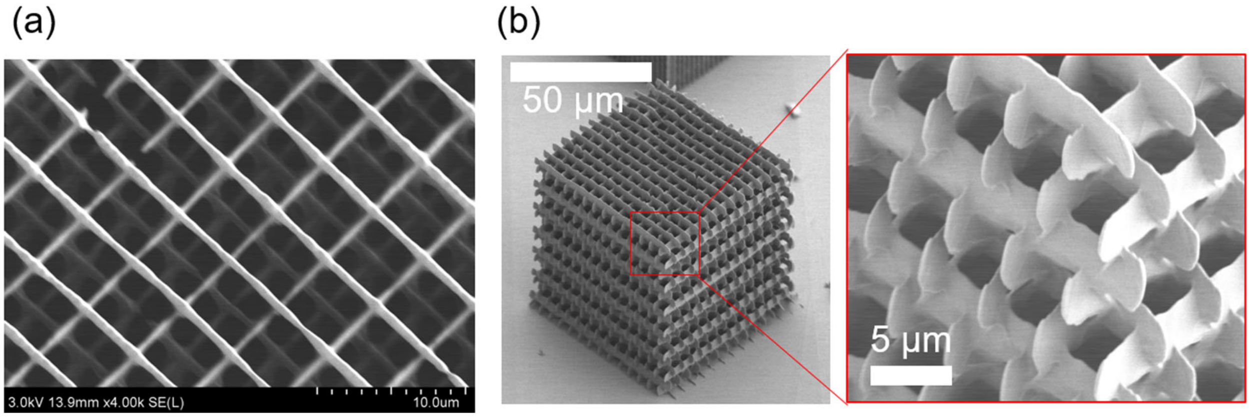

Figure S24: Demonstration of GP-TPL-based 3D printing of woodpiles with visible pores along the vertical surface. **a**, View from top showing wires in distinct layers. **b**, Side view showing distinct wires in the various layers and pores along the vertical surface.

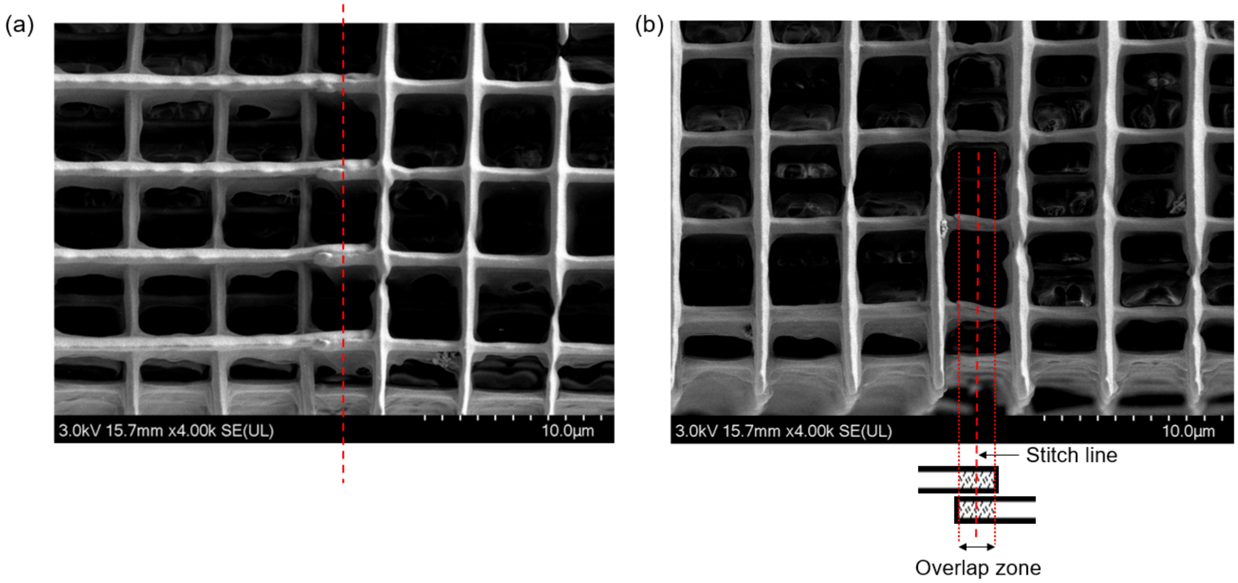

Figure S25: Demonstration of GP-TPL-based compensation of over-polymerization along voxel-level lap joints. Joints were produced by overlapping the line features along the horizontal axis over half the pitch. **a**, Lap joints produced by overlapping fully white motifs. **b**, Lap joints produced using grayscale masks that contained motif number 6 in the overlap region. The motif-based mask pattern that was used in the overlap region is shown below the SEM image.

(a)

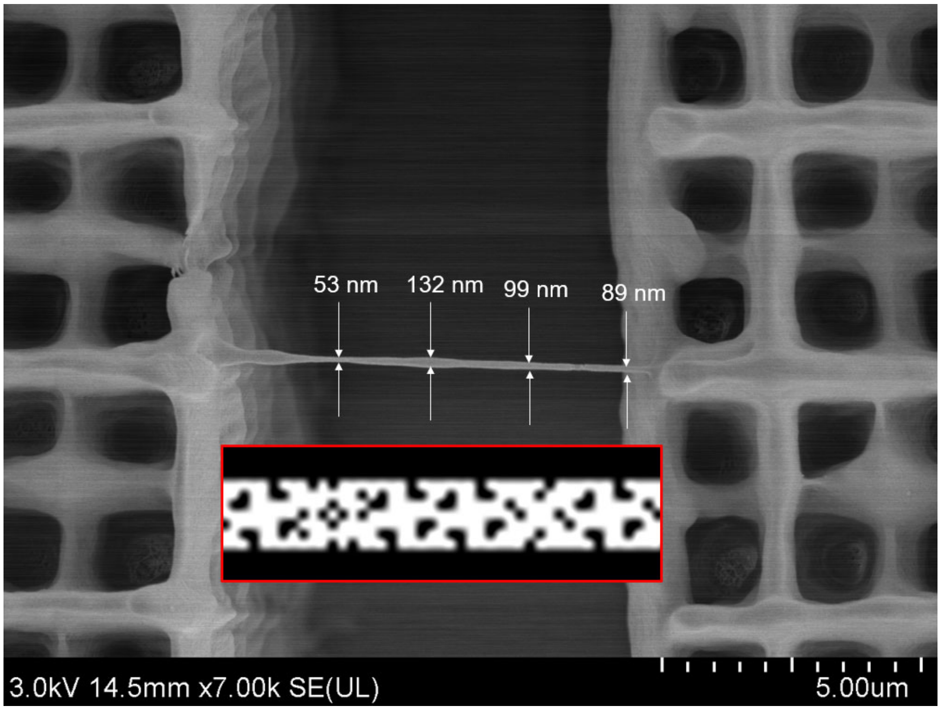

(b)

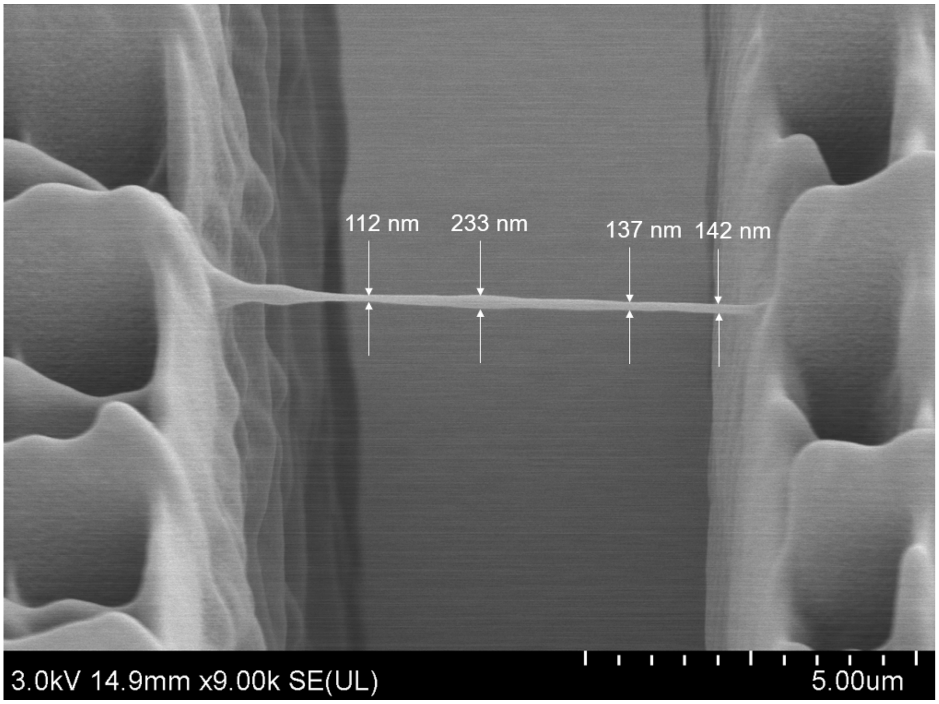

Figure S26: Demonstration of sub-100 nm feature size during printing of widely separated suspended nanowires. **a**, View from top. The motif-based mask pattern that was used to print the nanowire is overlaid on the image next to the nanowire. Motif number 9 was used in the thinnest section whereas the adjacent motifs were motif number 8. **b**, Side view at 45° tilt. Marked heights in **b** account for the correction due to the tilted view.

Table S4: Rates and resolutions of TPL implementations

#	Study	Printing modality	Benchmark structure	Minimum line width (nm)	Minimum pore size (nm)	Voxel generation rate (voxels/s)	Volumetric rate (mm^3/hr)
**1**	**This work**	**Projection**	**3D porous**	**269**	**509**	**7.9×10^7**	**217**
**2**	**This work (W)**	**Projection**	**Isolated nanowire**	**53**	-	**7.9×10^7 1.7×10^9**	**217**
3	S1(W)	Multi-beam, scanning	Isolated nanowire	400	-	9×10^6	1.15
4	S2(W)	Projection	Isolated nanowire	1000	-	8×10^3	0.6
5	S3(W)	Projection	Isolated nanowire	600	-	6.8×10^3	0.0087
6	S4(W)	Projection	Isolated nanowire	135	-	3.3×10^8	20
7	S4	Projection	3D porous	550	3080	3.3×10^8	20
8	S5(W)	Projection	Isolated nanowire	200	-	3.2×10^6	3.9
9	S5	Projection	3D porous	380	1670	3.2×10^6	3.9
10	S6(W)	Holography	Isolated nanowire	90	-	2×10^6	54
11	S6	Holography	3D porous	700	5090	2×10^6	54
12	S7	Projection	3D porous	285	650	7.6×10^5	0.8
13	S8	Holography	3D porous	500	1500	1×10^4	0.024
14	S9	Holography	3D porous	1000	4120	1.1	0.07
15	S10	Holography	3D porous	454	2580	1×10^2	3.9×10^{-4}
16	S11	Scanning	3D porous	100	1900	1×10^2	8.5×10^{-7}
17	S12	Scanning	3D porous	41	259	1.2×10^3	8.6×10^{-7}
18	S13	Scanning	3D porous	424	576	1.2×10^5	0.076
19	S14(W)	Holographic scanning	Isolated nanowire	215	-	1.5×10^8	0.11
20	S14	Holographic scanning	3D porous	1570	1667	1.5×10^8	0.11
21	S15(W)	Scanning	Isolated nanowire	475	-	1×10^8	4.56
22	S16(W)	Scanning – multi foci	Isolated nanowire	113	-	1×10^8	-

S12. Processing parameters for GP-TPL

Table S5: Processing parameters for GP-TPL

#	Figure #	Beam profile on DMD	Beam power	Exposure time (ms)	Photoinitiator concentration (wt %)	TEMPO concentration (ppm)
1	Fig. 3d	Flat	177 nW/pixel	13	0.25	0
2	Fig. 4a	Flat	177 nW/pixel	5	0.25	0
3	Fig. 4b	Flat	177 nW/pixel	1.4	0.25	0
4	Fig. 4c, left	Gaussian	-	2	0.25	0
5	Fig. 4c, right	Gaussian	-	3	0.25	0
6	Fig. 5a	Flat	216 nW/pixel	8	0.25	1000
7	Fig. 5b	Flat	216 nW/pixel	5	0.25	1000
8	Fig. 5c	Flat	216 nW/pixel	1	0.25	500
9	Fig. 5d, 5e	Flat	216 nW/pixel	Single pulse	1.0	0
10	Fig. S8a	Flat	177 nW/pixel	-	0.25	0
11	Fig. S8b	Flat	177 nW/pixel	13	0.25	0
12	Fig. S8c	Flat	177 nW/pixel	2	0.25	0
13	Fig. S8d	Flat	177 nW/pixel	20	0.25	0
14	Fig. S9b, d	Flat	177 nW/pixel	5	0.25	0
15	Fig. S10b, d	Flat	177 nW/pixel	1.4	0.25	0
16	Fig. S13b	Gaussian	-	2	0.25	0
17	Fig. S13d	Gaussian	-	3	0.25	0
18	Fig. S15b, d	Gaussian	-	8	0.25	0
19	Fig. S16, 2nd row	Gaussian	-	1.6	-	0
20	Fig. S16, 3rd row	Gaussian	-	1.8	-	0
21	Fig. S19b, d	Flat	216 nW/pixel	Single pulse	1.0	0
22	Fig. S20b, d	Flat	216 nW/pixel	Single pulse	1.0	0
23	Fig. S21c	Flat	216 nW/pixel	Single pulse	1.0	0
24	Fig. S23a	Flat	177 nW/pixel	-	0.25	0
25	Fig. S23b	Flat	216 nW/pixel	Single pulse	1.0	0
26	Fig. S24a, b	Flat	216 nW/pixel	5	0.25	1000
27	Fig. S25a, b	Flat	216 nW/pixel	5	0.25	1000
28	Fig. S26a, b	Flat	216 nW/pixel	1	0.25	500
