# Supplementary material for: Grayscale projection two-photon lithography using sub-diffraction motifs for ultrafast and precise nanoscale 3D printing
Source: Nat Commun. 2026 May 29;17:6981. doi: 10.1038/s41467-026-73782-x (PMC13392389; doi:10.1038/s41467-026-73782-x)
Supplement: Supplementary file 2 — Transparent Peer Review file [file 41467_2026_73782_MOESM2_ESM.pdf]

nature portfolio

Peer Review File

Grayscale projection two-photon lithography using sub-diffraction motifs for ultrafast and precise nanoscale 3D printing

Corresponding Author: Professor Sourabh Saha

This file contains all reviewer reports in order by version, followed by all author rebuttals in order by version.

Version 0:

Reviewer comments:

Reviewer #1

(Remarks to the Author)
In this manuscript, Kim and Saha present a grayscale projection two-photon lithography (GP-TPL) technique that enables deterministic voxel-level intensity tuning through sub-diffraction binary motifs. The authors demonstrate compensation of proximity effects, correction of non-uniform illumination, and high-speed 3D printing. Overall, the work is technically sound and experimentally comprehensive, and it represents a meaningful advance toward high-throughput nanoscale 3D fabrication. We therefore recommend minor revision prior to acceptance in Nature Communications.

1. The physical mechanism underlying the selection of the 32 motifs (25 sets) should be more clearly explained. Specifically, although the authors state that these motifs have nearly identical FWHM, the criteria used to exclude the other 8160 symmetric patterns remain insufficiently described. Additional discussion on the robustness of motif intensity ordering and its dependence on optical aberrations would strengthen the work.

2. In Fig. 1d, the printed structure produced with fully white grating lines exhibits a cone-shaped over-polymerized region near the top. Could the authors clarify the physical origin of this cone formation? Additionally, is the over-polymerization confined only to the uppermost layers, or does it influence deeper regions of the 3D structure as well?

3. In Fig. 3b, the motif-number range varies for different exposure times, and some exposure conditions do not include all 32 motifs. Could the authors explain why the complete motif library was not tested for each exposure time?

4. The compensation of proximity effects is convincing, but the proximity-effect compensation is demonstrated only on periodic woodpile structures. Could the authors clarify whether the motif-based grayscale method also applies to non-periodic or other geometries?

5. Please add the labels for “uncompensated” and “compensated” printing in Figure 4, as described in the captions.

6. In the motif pattern, what is smallest pixel size that can be achieved? Also, what happens if the number of pixels is reduced to 3 x 3 (i.e. can the intensity still be tuned effectively)?

7. What could be some drawbacks of using a single fs laser pulse in GP-TPL, as opposed to using multiple pulses?

8. The authors fixed the exposure time of the fs laser pulse. Is there any benefit in varying the pulse exposure time between layers?

9. The authors may also find it helpful to consider relevant work in the area of nanoscale 3D-printed diffractive optics, such as: https://doi.org/10.1038/s41467-021-23964-6; https://doi.org/10.1002/adom.201900068, and https://doi.org/10.1002/adfm.202214211.

Reviewer #2

(Remarks to the Author)
This manuscript presents a highly innovative GP-TPL technique that resolves the longstanding speed-accuracy trade-off in nanoscale 3D printing. By leveraging sub-diffraction binary motifs to achieve grayscale intensity control without sacrificing resolution, the method significantly enhances both printing efficiency and quality. The authors have thoroughly validated their approach across multiple challenging scenarios, including proximity effect correction, beam profile non-uniformity compensation, and single-pulse-per-layer printing, demonstrating its broad applicability and robustness. I believe this work meets the high standard for publication in Nature Communications after the authors address the following points:
1. Figure 6a appears to contain a minor layout error (stray letter 'b').
2. Would the motif design require recalibration for different optical configurations? For example, if the DMD were replaced with another model.
3. As the method's performance may depend on the photopolymer resist's sensitivity and nonlinear response to light intensity, could the authors comment on how variations in resist properties might influence motif selection?
4. The demonstrated structures appear to be fabricated within a single exposure field. Have the authors explored large-area patterning using field stitching? If so, could grayscale compensation be applied to mitigate artifacts at stitching boundaries?
5. While woodpile structures effectively illustrate the key capabilities of GP-TPL, could additional examples be provided to better showcase its versatility and practical potential?

Reviewer #3

(Remarks to the Author)
In this manuscript, the authors present grayscale-projection two-photon lithography (TPL) using a digital micromirror device (DMD). They employ sub-diffraction binary motifs to generate controlled grayscale intensity levels (32 levels) and investigate exposure-dose control through these motifs. Based on this approach, the authors claim high-fidelity projection printing with single-pulse-per-layer exposure, achieving printing rates exceeding 75 million voxels per second and more than 200 mm3 per hour, with feature sizes below 300 nm. They further state that the performance of GP-TPL for fabricating porous 3D structures surpasses that of other TPL implementations in both speed and resolution.

It should be noted that DMD-based TPL for accelerating printing has been reported extensively in recent years, either in projection mode (https://doi.org/10.1038/s41377-021-00645-z, https://doi.org/10.1016/j.addma.2025.104929), including prior work by the corresponding author (https://doi.org/10.1126/science.aax8760, https://doi.org/10.1080/17452759.2023.2230979), or via multi-focus approaches enabling parallel writing (https://doi.org/10.1038/s467-019-10249-2, https://doi.org/10.1038/s41467-023-37163-y, https://doi.org/10.1364/OE.483524). Additional approaches for enhancing TPL printing speed can also be found in https://doi.org/10.1002/lpor.202301312. As such, the fundamental concept of using DMDs for TPL is not new.

Grayscale modulation in micro- and nanoscale 3D printing using nonlinear processes with DMDs has likewise been demonstrated previously, for example, by Somer et al. (https://doi.org/10.1038/s41377-021-00645-z, https://doi.org/10.1002/lpor.202500187), where it has been used to reduce proximity effects (https://doi.org/10.1002/lpor.202500187). In fact, reducing proximity effects in TPL is a well-known challenge that has been addressed by several exposure-optimization strategies (https://doi.org/10.1016/j.addma.2021.102491, https://doi.org/10.1016/j.addma.2025.104902), with notable contributions by the corresponding author in projection-based TPL printing (https://doi.org/10.1080/17452759.2023.2230979). Still, the use of binary motifs to control exposure dose is an interesting addition that could broaden the applicability of projection-based TPL. However, while the investigation of exposure-dose control appears sound, the printing results presented in the manuscript are currently underwhelming. Overall, the manuscript is, in its present form, somewhat misleading in several respects, making it difficult to clearly identify the novelty the authors intend to highlight.

- Considering the title of the manuscript, I cannot identify true nanoscale 3D-printed features of the type typically associated with TPL. Most structures appear bulky in the axial direction. It would be important for the authors to clarify the origin of this effect and whether it can be improved. For instance, spatio-temporal beam shaping has been shown to help achieve rounder voxel geometries (https://doi.org/10.29026/oea.2023.230066), which may be relevant here. The only structure that does not appear completely bulky is the buckyball, although its pores are on the micrometer scale rather than the nanoscale. Even for the woodpile structures, the lateral pore size is in the micrometer regime. While the authors report linewidths below 300 nm, the woodpile sidewalls are fully enclosed, which prevents classification of the results as nanoscale 3D printing. Could the quality of the structures be improved by using hybrid organic–inorganic polymers with low-shrinkage behavior and tailored photoinitiator systems, such as those reported in https://doi.org/10.1021/acsaom.4c00241 or https://doi.org/10.1021/acsapm.5c01802?

- The term “high fidelity” appears misleading. High fidelity typically implies well-defined, refined structural features. However, the structures shown, particularly in Fig. 5b, are of noticeably lower quality than what is commonly achieved with TPL. While reduced fidelity is an expected limitation of projection-based approaches, previously published porous 3D structures (https://doi.org/10.1080/17452759.2023.2230979) by the same corresponding author exhibit comparable quality to those presented here. Therefore, from the standpoint of structural fidelity alone, I do not observe a clear improvement.

- Although the title focuses on TPL, the manuscript extends beyond this scope by presenting results on ablating a thin gold layer using the same technique. This direction is fundamentally different from nanoscale 3D printing and is not reflected in the title, which may be misleading for readers and obscures the intended focus of the work.

- The true advantage of grayscale TPL lies in achieving high-fidelity structuring, which is essential for the fabrication of advanced micro-optics. This has been demonstrated using commercial TPL systems (https://doi.org/10.1364/OE.480472), and grayscale TPL can likewise be implemented in home-built setups using rapid laser-power modulation via an AOM (https://doi.org/10.37188/lam.2024.019). Since grayscale TPL is already established, it should be acknowledged in the introduction to avoid misleading statements about the novelty of the approach. It should also be considered to include examples from optics, where the use of grayscale TPL clearly provides an advantage, and to fully characterize the resulting optical elements. Such demonstrations would more convincingly highlight the practical relevance and benefits of the proposed approach.

- The authors intend to present woodpile structures in relation to their analysis on achievable linewidth and pore size; however, the only clear examples of woodpiles are the fully 3D structures shown in the main text (Fig. 3d) and in the supplementary material (e.g., Fig. S7 and Fig. S17d). The remaining examples more closely resemble 2D grid patterns stacked vertically rather than genuine woodpiles. Moreover, Figure S7a shows that even with grayscale control, proximity effects remain significant, with central regions exhibiting clear over-polymerization. It also appears that woodpile features of comparable quality can be achieved without grayscale control, raising questions about the added value of the proposed approach for this specific structure type.

- The authors aim to demonstrate flexibility by using two lenses and state that grayscale-projection TPL can be generalized to various optics. However, switching from a 60x NA 1.3 lens to a 40x NA 1.25 lens mainly affects the field of view; the focusing properties are only marginally different. It would be useful to clarify whether dry objective lenses with NA values close to 1 can also be used.

- The claim of exceeding 75 million voxels/s and >200 mm3/h with <300 nm features is highly sensitive and requires careful contextualization beside the benchmark analysis provided. Printing rate depends strongly on setup design, resist properties, target geometry, and other parameters. Somers et al. (https://doi.org/10.1038/s41377-021-00645-z) already achieved ~10^8voxels/s using a 100x lens, and the manuscript itself notes that lower-magnification lenses increase throughput. Parallel-scanning DMD-TPL systems have achieved even higher printing rates, ranging from 10^8 voxels/s (https://pubs.acs.org/doi/10.1021/acs.nanolett.4c00505) to 10^12 voxels/s (https://www.light-am.com/article/doi/10.37188/lam.2024.003). Neither of these works is referenced in the manuscript, despite their direct relevance.

Version 1:

Reviewer comments:

Reviewer #1

(Remarks to the Author)
The authors have addressed all concerns and the manuscript is ready for publication

Reviewer #2

(Remarks to the Author)
The authors have addressed my previous questions well. I have no further comments and recommend the manuscript for publication.

Reviewer #3

(Remarks to the Author)
The authors have thoroughly addressed the concerns and significantly improved the manuscript. I recommend publication without any further modifications.

Open Access This Peer Review File is licensed under a Creative Commons Attribution 4.0 International License, which permits use, sharing, adaptation, distribution and reproduction in any medium or format, as long as you give appropriate credit to the original author(s) and the source, provide a link to the Creative Commons license, and indicate if changes were made.
In cases where reviewers are anonymous, credit should be given to 'Anonymous Referee' and the source.
The images or other third party material in this Peer Review File are included in the article's Creative Commons license, unless indicated otherwise in a credit line to the material. If material is not included in the article's Creative Commons license and your intended use is not permitted by statutory regulation or exceeds the permitted use, you will need to obtain permission directly from the copyright holder.
To view a copy of this license, visit https://creativecommons.org/licenses/by/4.0/

Authors’ response to reviewers

The authors thank the reviewers for their comments. Your responses were very helpful in improving the content and presentation of this manuscript. Please find our responses to your comments included herein. Our changes in the manuscript are listed below and highlighted in yellow in the revised manuscript.

Reviewer #1:

In this manuscript, Kim and Saha present a grayscale projection two-photon lithography (GP-TPL) technique that enables deterministic voxel-level intensity tuning through sub-diffraction binary motifs. The authors demonstrate compensation of proximity effects, correction of non-uniform illumination, and high-speed 3D printing. Overall, the work is technically sound and experimentally comprehensive, and it represents a meaningful advance toward high-throughput nanoscale 3D fabrication. We therefore recommend minor revision prior to acceptance in Nature Communications.

We thank the reviewer for this favorable assessment of our work. We have provided responses to the reviewer’s specific comments below.

1. The physical mechanism underlying the selection of the 32 motifs (2^5 sets) should be more clearly explained. Specifically, although the authors state that these motifs have nearly identical FWHM, the criteria used to exclude the other 8160 symmetric patterns remain insufficiently described. Additional discussion on the robustness of motif intensity ordering and its dependence on optical aberrations would strengthen the work.

We selected the 32 motifs by first identifying those motifs that have the same nominal size as the fully white motif (as shown in Fig. S7(a)) and have intensity between 10% to 100%. This reduced the feasible set to 711 motifs (as shown in the newly added Fig. S7(b)). Then, we identified motifs that progressively reduced the intensity value by approximately 7.7%, i.e., the next motif was selected to be the one that reduced the intensity from the previous level by 7.7%. We have updated the text in Sec. 2 (4th paragraph) to summarize our approach for motif selection. With the 7.7% step size we found the ordering of the motifs to be robust (i.e., the ordering does not change) under optical aberrations, as shown in the newly added Fig. S5(c). However, the ordering may not remain robust with finer steps. We intend to further study this aspect in a follow-on work focused on identification of motifs for finer grayscale levels. Within the context of this work, the 7.7% step size is fine enough to demonstrate dosage control on the sub-100 nm length scale (as shown in the newly added Figs. 5(c) and S26).

2. In Fig. 1d, the printed structure produced with fully white grating lines exhibits a cone-shaped over-polymerized region near the top. Could the authors clarify the physical origin of this cone formation? Additionally, is the over-polymerization confined only to the uppermost layers, or does it influence deeper regions of the 3D structure as well?

As described in the caption of Fig. 1, this structure was printed with a non-uniform Gaussian beam. The over-polymerized region corresponds to the section of the beam that has the highest intensity. The structure shown in Fig. 1(d) is the same structure that was compensated later in Fig. 4(c). As we discussed the origin of this defect and its compensation in detail later with the rest of the data summarized in Fig. 4, we did not make any changes to Fig. 1 in our revised manuscript. We briefly edited the caption of Fig. 1 to highlight the presence/absence of defects.

Over-polymerization due to non-uniform Gaussian beam occurs even in deeper regions of the structure as it is driven by non-uniform intensity distribution of the beam. For example, even with one layer of exposure, we observe non-uniform processing in ablated features (Fig. 7(c)). When over-polymerization occurs due to proximity effects, we observe that it scales with the height of the structure (as shown in the newly added Fig. 5(a), top row). Deeper regions (that were printed first) may not exhibit any over-polymerization but the outer layers (i.e., those on the top) exhibit over-polymerization. This observation is consistent with the expectation that proximity effects are higher in denser architectures.

3. In Fig. 3b, the motif-number range varies for different exposure times, and some exposure conditions do not include all 32 motifs. Could the authors explain why the complete motif library was not tested for each exposure time?

For Fig. 3b, we performed experiments with a larger set of motifs than shown in this figure. For higher numbered motifs that are not shown in this figure, we observed that the desired woodpile structures could not be produced due to the low dosage values. For very low exposures, the intensity of higher-numbered motifs is too low to produce the 3D structures. These regimes correspond to the left lower quadrant in the supplementary image Fig. S8 (a). The woodpiles in this region were either only partially printed or not printed at all. We have updated Fig. S8(a) by marking the parameters that were varied along the X and Y axes. We have also edited the text in the main manuscript (paragraph above Fig. 3) to highlight that some motifs do not appear on the plot because the dosage was too low to produce the woodpiles.

4. The compensation of proximity effects is convincing, but the proximity-effect compensation is demonstrated only on periodic woodpile structures. Could the authors clarify whether the motif-based grayscale method also applies to non-periodic or other geometries?

Yes, our motif-based grayscale method can be also be applied to non-periodic geometries. For example, in our original submission, we demonstrated the compensation of proximity-effects in non-periodic geometries. Specifically, Fig. 5(b) from the original submission (renumbered as Fig. 5(e) in the revised version) compares the fidelity of uncompensated and compensated printing for a solid cylinder. The excessive uncontrolled printing in the left panel is due to proximity effects that arise in dense architectures. As shown in the bottom panel, this excessive printing was suppressed by applying the motif-based grayscale mask. In our revised manuscript, we have provided additional examples of compensation in voxel-level geometries, such as suspended nanowires and lap joints (newly added Figs. 5(b) and (c)).

5. Please add the labels for "uncompensated" and "compensated" printing in Figure 4, as described in the captions.

We have updated Fig. 4 with appropriate labels to identify uniform vs. grayscale masks.

6. In the motif pattern, what is smallest pixel size that can be achieved? Also, what happens if the number of pixels is reduced to 3 x 3 (i.e. can the intensity still be tuned effectively)?

Although smaller motifs can produce smaller spots, their use in grayscale control is limited by the tradeoffs. The number of grayscale levels decreases as the footprint of the motif decreases. For example, a single pixel is the smallest possible motif footprint, but it can only have two levels (0 or 1). In theory, a motif with 3×3 footprint can have a total of 2^9 grayscale levels. However, in practice, many of these motifs will produce asymmetric spots and spots of various sizes. The set of desired motifs (i.e., symmetric with near-constant size) will be smaller, which will limit the number of practical grayscale levels. A more significant drawback is that motifs smaller than the diffraction limit (i.e., 7×7 pixels with the 60× lens) demonstrate a significant drop in intensity. This is because a larger fraction of the intensity is filtered out by the lens in the sub-diffraction regime. For example, a fully-bright 3×3 motif has peak intensity that is 0.12 times the peak intensity of a fully bright 7×7 motif. As absorption scales nonlinearly with intensity in TPL, printing with smaller motifs becomes extremely challenging. For example, the motif intensity may not be high enough to print while the intensity incident on the DMD may exceed its damage threshold. Working at the resolution limit (i.e., 7×7 footprint) provides a balance with respect to intensity and spot size. We have updated Fig. S2 by adding the data for the 3×3 motif and the ratio of peak intensities with motifs of various side lengths. Additionally, in our revised manuscript, we have demonstrated the printing of voxels as thin as 53 nm with the 7×7 motifs (Fig. 5(c)). Thus, having a large motif footprint does not prevent us from achieving sub-100 nm features.

7. What could be some drawbacks of using a single fs laser pulse in GP-TPL, as opposed to using multiple pulses?

As discussed in our original submission, the major drawback of using a single fs laser pulse is that the design space is significantly smaller. Specifically, the achievable dosages and dosage distributions in each layer become constrained to a single uniform dosage. For many geometries, such those with densely packed features, the lack of dosage tunability makes it impossible to print the desired structures (i.e., structures without excessive uncontrolled polymerization in the center).

When multiple pulses are used to write each layer, the dosage can be finely tuned (by changing the number of pulses). Additionally, the dosage in different regions of the same layer can be non-uniformly varied by using multiple masks, as demonstrated in our past work (Reference 37). Although the multiple exposure approach enables extensive tuning of dosage, it comes at a throughput tradeoff because one must wait for multiple pulses to arrive. When a single fs pulse is used per layer, it is impossible to apply existing grayscale approaches that rely on multi-pulse exposure. GP-TPL overcomes this fundamental constraint by enabling deterministic voxel-level control of dosage over multiple levels **using a single fs pulse**. This enables printing significantly faster (at 0.2 ms per layer instead of >10 ms per layer). Our data summarized in Fig. 5(e) highlights this specific advantage of GP-TPL. When we attempted rapid printing of a solid cylinder with a single fs pulse per layer, we observed excessive uncontrolled polymerization (top panel). The excessive printing was suppressed ***while rapidly printing with a single fs pulse per layer*** by applying a motif-based grayscale mask (bottom panel). Other grayscale techniques, which are based on multiple pulses per layer, cannot preserve the rapid printing speeds. Furthermore, GP-TPL can be also be applied with multiple pulses per layer to further improve the fidelity of printing without sacrificing throughput, as demonstrated in Fig. 4(b). In our previous work (Reference 37), we overcame the proximity effects by using multiple masks but it lowered the throughput (by 3×). **Here, with GP-TPL, we overcame proximity effects without sacrificing the throughput**. **Additionally, GP-TPL enabled us to demonstrate rapid continuous axial-scanning printing at a significantly higher rate (this study vs. marker S7 in Fig. 6).**

8. The authors fixed the exposure time of the fs laser pulse. Is there any benefit in varying the pulse exposure time between layers?

Yes, in multi-pulse exposure schemes, varying the exposure between layers provides the benefit of wider tunability of dosage. As proximity effects increase with increasing height of structures, this additional tunability can be advantageously leveraged to suppress proximity effects and improve fidelity. However, the tradeoff is that this approach makes it difficult to synchronize the Z-motion with the different DMD exposures. Furthermore, multi-pulse exposure techniques are fundamentally rate-limited as they rely on multiple pulses to arrive. GP-TPL enables suppressing proximity effects without changing the exposure between the layers (as demonstrated in the newly added Fig. 5(a)) and also while using a single fs pulse per layer.

9. The authors may also find it helpful to consider relevant work in the area of nanoscale 3D-printed diffractive optics, such as: https://doi.org/10.1038/s41467-021-23964-6; https://doi.org/10.1002/adom.201900068, and https://doi.org/10.1002/adfm.202214211.

Thank you for suggesting these articles. We have included them in the introduction section of our revised manuscript to highlight the optical applications of TPL.

Reviewer #2:

This manuscript presents a highly innovative GP-TPL technique that resolves the longstanding speed-accuracy trade-off in nanoscale 3D printing. By leveraging sub-diffraction binary motifs to achieve grayscale intensity control without sacrificing resolution, the method significantly enhances both printing efficiency and quality. The authors have thoroughly validated their approach across multiple challenging scenarios, including proximity effect correction, beam profile non-uniformity compensation, and single-pulse-per-layer printing, demonstrating its broad applicability and robustness. I believe this work meets the high standard for publication in Nature Communications after the authors address the following points:

We thank the reviewer for this favorable assessment of our work. We have provided responses to the reviewer's specific comments below.

1. Figure 6a appears to contain a minor layout error (stray letter 'b').

Thank you for pointing out this error. We have fixed this in our revised manuscript.

2. Would the motif design require recalibration for different optical configurations? For example, if the DMD were replaced with another model.

Yes, the motif designs must be recalibrated if certain combinations of parameters in the optical configuration are changed. Specifically, if the number of pixels that fit within the diffraction-limited spot size changes, then the motifs must be recalibrated. Nevertheless, the motif-based grayscale approach remains valid even with different optical configurations. In our original submission, we demonstrated the generalizability of our grayscale approach by calibrating the motifs for two different optical configurations: (a) 60× objective lens (7 pixels fit within the diffraction-limited spot) and (b) 40× objective lens (5 pixels fit within the diffraction-limited spot). In both configurations, we have demonstrated the ability to use motifs to implement grayscale dosage control.

For the specific case of change in DMD model, the motif designs will not require recalibration if the pixel pitch of the DMD remains unchanged. If the pixel pitch changes, the same motif design can be preserved by increasing/decreasing the magnification of the projection system by changing the focal length of the collimating lens. Doing so will preserve the number of pixels (in the image plane) within the footprint of the diffraction-limited spot size. We have edited the text in Sec. 2 (2nd last paragraph) to include this discussion.

3. As the method's performance may depend on the photopolymer resist's sensitivity and nonlinear response to light intensity, could the authors comment on how variations in resist properties might influence motif selection?

During compensation of non-uniform light intensify distribution in the incoming beam (such as for a Gaussian beam shape), we do not expect any change in the motif selection based on different resists. This is supported by the evidence provided in our original submission (Fig. 4(c) and Fig. 7(c)), wherein we compensated non-uniformity during printing and ablation due to a Gaussian beam.

During compensation of over-polymerization due to proximity effects, one would generally expect the motif selection to be dependent upon the resist's sensitivity. To test this, we performed additional experiments with resists of various sensitivities (i.e., with resists containing more versus less quencher). Our experiments demonstrate that the same motif selection is effective in suppressing over-polymerization with various resists (shown in newly added Fig. S17). It is noteworthy that the exposure (i.e., net dosage) at which over-polymerization occurs differ for the different resists due to their different sensitivities. However, the spatial distribution of over-polymerization is still similar due to the identical feature densities. Thus, the same motif patterns were effective in suppressing over-polymerization with the different resists. We have edited the text in Sec. 2 (7th paragraph) to highlight the generalizability of our motif selection with respect to the resist's sensitivity.

4. The demonstrated structures appear to be fabricated within a single exposure field. Have the authors explored large-area patterning using field stitching? If so, could grayscale compensation be applied to mitigate artifacts at stitching boundaries?

Yes, we have explored large-area patterning using field stitching, but we have not reported those results here because our stitching errors are dominated by the errors in the motion stages. There are generally two major sources of error during field stitching: (1) due to misalignment arising from motion errors and (2) due to excessive polymerization near the edges of the fields. GP-TPL is effective in suppressing the stitching defects arising from the 2nd factor. We have provided additional data (shown in Figs. 5(b) and S25) to demonstrate how GP-TPL can enable high-quality lap joints by suppressing over-printing in the overlap region of the stitches.

5. While woodpile structures effectively illustrate the key capabilities of GP-TPL, could additional examples be provided to better showcase its versatility and practical potential?

In our original submission, we had provided additional examples of structures such as Bucky ball and solid cylinder (numbered Figs. 5(d) and (e) in the revised version). In our revised submission, we have provided the following additional examples to highlight the versatility and potential of GP-TPL.

(a) compensation on the scale of a single nanowire demonstrating the potential of GP-TPL in reducing the minimum linewidth from >100 nm to as small as 53 nm (Fig. 5(c)) and reduce the line height from > 1000 nm to ~ 100 nm.

(b) compensation of over-printing in the overlap zone of voxel-level lap joints during field stitching (Fig. 5(b)).

(c) high-fidelity woodpiles with porosity along the vertical surfaces produced by applying grayscale masks (Figs. 5(a) and S24).

Reviewer #3:

In this manuscript, the authors present grayscale-projection two-photon lithography (TPL) using a digital micromirror device (DMD). They employ sub-diffraction binary motifs to generate controlled grayscale intensity levels (32 levels) and investigate exposure-dose control through these motifs. Based on this approach, the authors claim high-fidelity projection printing with single-pulse-per-layer exposure, achieving printing rates exceeding 75 million voxels per second and more than 200 mm3 per hour, with feature sizes below 300 nm. They further state that the performance of GP-TPL for fabricating porous 3D structures surpasses that of other TPL implementations in both speed and resolution.

1. It should be noted that DMD-based TPL for accelerating printing has been reported extensively in recent years, either in projection mode (https://doi.org/10.1038/s41377-021-00645-z, https://doi.org/10.1016/j.addma.2025.104929), including prior work by the corresponding author (https://doi.org/10.1126/science.aax8760, https://doi.org/10.1080/17452759.2023.2230979), or via multi-focus approaches enabling parallel writing (https://doi.org/10.1038/s467-019-10249-2, https://doi.org/10.1038/s41467-023-37163-y, https://doi.org/10.1364/OE.483524). Additional approaches for enhancing TPL printing speed can also be found in https://doi.org/10.1002/lpor.202301312. As such, the fundamental concept of using DMDs for TPL is not new.

We agree with the reviewer that DMD-based TPL is not novel. We did not claim novelty on the concept of using DMDs for TPL. **Our novelty lies in enabling direct and independent control of intensity at each focal spot within the entire field while using binary DMD-based masks and a single pulse of fs laser for image projection**. This capability enables high-speed TPL printing, compensation of non-uniform over-polymerization, and fine control of dosage on the voxel level.

2. Grayscale modulation in micro- and nanoscale 3D printing using nonlinear processes with DMDs has likewise been demonstrated previously, for example, by Somer et al. (https://doi.org/10.1038/s41377-021-00645-z, https://doi.org/10.1002/lpor.202500187), where it has been used to reduce proximity effects (https://doi.org/10.1002/lpor.202500187). In fact, reducing proximity effects in TPL is a well-known challenge that has been addressed by several exposure-optimization strategies (https://doi.org/10.1016/j.addma.2021.102491, https://doi.org/10.1016/j.addma.2025.104902), with notable contributions by the corresponding author in projection-based TPL printing (https://doi.org/10.1080/17452759.2023.2230979). Still, the use of binary motifs to control exposure dose is an interesting addition that could broaden the applicability of projection-based TPL. However, while the investigation of exposure-dose control appears sound, the printing results presented in the manuscript are currently underwhelming. Overall, the manuscript is, in its present form, somewhat misleading in several respects, making it difficult to clearly identify the novelty the authors intend to highlight.

Our grayscale approach is fundamentally different from past dose control approaches in TPL that use multiple pulses because it enables **direct and independent control of *intensity* at each focal point over the entire field**. It does not rely on controlling the duration of exposure to achieve the grayscale effect. This key novelty of our approach enables achieving grayscale control of dose **even with a single pulse of femtosecond light**. This is a significant advance over the state-of-art because it enabled us to push the rate of projection-based printing to its theoretical limit. Specifically, the rapid continual Z-axis scanning based printing with one fs pulse per layer, which we demonstrated (Fig. 5(d) and (e)), cannot be achieved without direct control of intensity. Furthermore, due to the direct intensity control, our approach enables **resist-agnostic compensation of proximity effects**, as demonstrated through additional experiments summarized in Fig. S17. We believe that the structures in our original submission supported the novelty of our approach. Nevertheless, we have printed additional structures to highlight the novelty of GP-TPL in terms of **enabling fine control of dosage at the voxel level** to

achieve a minimum feature size <**60 nm** (Fig. 5(c)). Our original manuscript already cited the first reference (Somer et al.), third reference (Arnoux et al.), and the fifth reference (our past work) at relevant places. In the revised manuscript, we have included all five references in the Introduction section within the context of discussion of DMD-based techniques and proximity effects.

3. Considering the title of the manuscript, I cannot identify true nanoscale 3D-printed features of the type typically associated with TPL. Most structures appear bulky in the axial direction. It would be important for the authors to clarify the origin of this effect and whether it can be improved. For instance, spatio-temporal beam shaping has been shown to help achieve rounder voxel geometries (https://doi.org/10.29026/oea.2023.230066), which may be relevant here. The only structure that does not appear completely bulky is the buckyball, although its pores are on the micrometer scale rather than the nanoscale. Even for the woodpile structures, the lateral pore size is in the micrometer regime. While the authors report linewidths below 300 nm, the woodpile sidewalls are fully enclosed, which prevents classification of the results as nanoscale 3D printing. Could the quality of the structures be improved by using hybrid organic–inorganic polymers with low-shrinkage behavior and tailored photoinitiator systems, such as those reported in https://doi.org/10.1021/acsaom.4c00241 or https://doi.org/10.1021/acsapm.5c01802?

In our original submission, we focused on printing bulky structures because these tend to be more challenging to print with P-TPL due to the presence of higher proximity effects in denser geometries. To highlight the nanoscale resolution of our technique, in our revised manuscript, we have demonstrated GP-TPL-based printing of:

(a) Suspended **nanowires as thin as 53 nm** along the width and **112 nm along the height** (Figs. 5(c) and S26). Fine control of intensity, enabled by GP-TPL, allowed us to finely control the nanowire width and reduce the heights from > 1 µm to ~100 nm.

(b) Woodpiles with **distinct porosity** along the height direction. The nanowires in the various layers can be distinguished in the top and side views (Figs. 5(a) and S24).

4. The term "high fidelity" appears misleading. High fidelity typically implies well-defined, refined structural features. However, the structures shown, particularly in Fig. 5b, are of noticeably lower quality than what is commonly achieved with TPL. While reduced fidelity is an expected limitation of projection-based approaches, previously published porous 3D structures (https://doi.org/10.1080/17452759.2023.2230979) by the same corresponding author exhibit comparable quality to those presented here. Therefore, from the standpoint of structural fidelity alone, I do not observe a clear improvement.

It is unfair to compare the fidelity of Fig. 5(b) (numbered Fig. 5(e) in the revised manuscript) with our previous work because our previous work was performed under less extreme processing conditions. The structure shown in Fig. 5(e) was printed with **a single-pulse per layer and rapid continuous axial scanning**. This is an extreme condition for proximity effects and this condition severely limits dosage tunability (due to lack of exposure time as a controllable variable). **To the best of our knowledge, there have been no other demonstration of 3D printing (of any quality) with a single femtosecond pulse per layer and continuous axial scan.** To highlight the fidelity improvement due to GP-TPL, in our original submission, we benchmarked our improvements by showing images with and without grayscale masks (top versus bottom panel in Fig. 5(e)). Without grayscale masks, severe uncontrolled over-polymerization is observed over the cylinder to the extent that it is impossible to stack a lattice with well-defined cuboid geometry on top of the cylinder (top panel). Grayscale masks suppress this over-printing (bottom panel)

Nevertheless, we appreciate the reviewer's concern that high-fidelity should mean well-defined structures. Therefore, in our revised manuscript, we have provided images of newly printed woodpiles with well-defined and refined features to highlight the high-fidelity of GP-TPL. These woodpiles were

printed under less extreme processing conditions than those for Fig. 5(e) and under conditions that are similar to our past work (i.e., with multiple pulses per layer and without axial scanning). The woodpile shown in Fig 5(a) (bottom row) is of distinctly higher quality than our previous work. Specifically, shrinkage is minimal, nanowires are well defined, and the features are uniform over the entire field. Therefore, we believe that our data supports our claim that GP-TPL provides a distinct improvement in the fidelity of projection-based TPL.

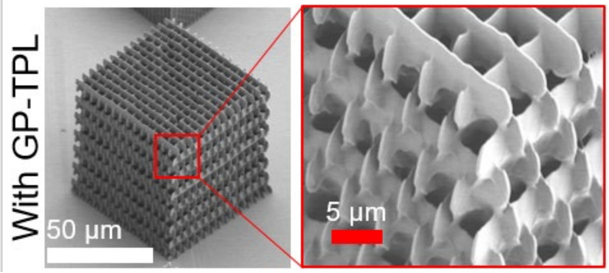	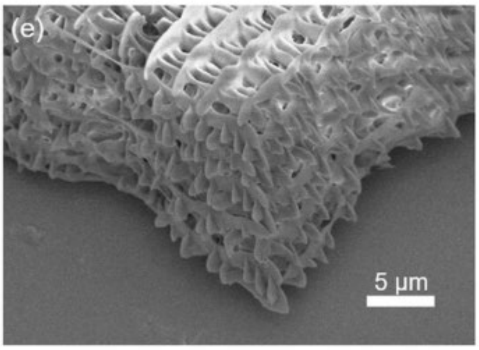
Woodpile produced with GP-TPL in the current work	Woodpile from our past work (Reference 37)

5. Although the title focuses on TPL, the manuscript extends beyond this scope by presenting results on ablating a thin gold layer using the same technique. This direction is fundamentally different from nanoscale 3D printing and is not reflected in the title, which may be misleading for readers and obscures the intended focus of the work.

As discussed in Sec. 2 (last paragraph), the purpose of Fig. 7(c) (with ablation results) is to **highlight the generalizability** of our motif-based grayscale approach. The ablation demonstration highlights that our grayscale approach does not rely on knowledge about the resist's optical response; instead, it can directly and independently control the intensity of the voxels. To reduce ambiguity, in this paragraph, we have replaced "GP-TPL" with "motif-based grayscale approach" to highlight that we are demonstrating the generalizability of the motif-based approach. We prefer to keep this figure in the main manuscript, but will be happy to move it to the supplementary material if the editor suggests so based on scope or length considerations.

6. The true advantage of grayscale TPL lies in achieving high-fidelity structuring, which is essential for the fabrication of advanced micro-optics. This has been demonstrated using commercial TPL systems (https://doi.org/10.1364/OE.480472), and grayscale TPL can likewise be implemented in home-built setups using rapid laser-power modulation via an AOM (https://doi.org/10.37188/lam.2024.019). Since grayscale TPL is already established, it should be acknowledged in the introduction to avoid misleading statements about the novelty of the approach.

We had already mentioned in the Introduction section that AOM (i.e., acousto-optic modulator) can be used for grayscale control of dosage in scanning-based techniques (Reference 33). As we discussed in our original manuscript, this AOM-based approach **cannot independently tune the intensity of each focal spot** when multiple focal spots are produced by diffractively splitting the same beam into multiple beamlets. We thank the reviewer for providing these additional references; we have included the first reference in the Introduction section within the context of grayscale TPL. We did not include the second reference as it is focused more on improving the performance of microscopy than the performance of TPL.

7. It should also be considered to include examples from optics, where the use of grayscale TPL clearly provides an advantage, and to fully characterize the resulting optical elements. Such demonstrations would more convincingly highlight the practical relevance and benefits of the proposed approach.

We appreciate the reviewer's comment that grayscale TPL provides an advantage in printing optics. However, our work demonstrates that projection-based grayscale TPL with direct control of intensity **also enables achieving unprecedented speed and resolution while improving fidelity.** With additional data shown in the revised manuscript, we have demonstrated that nanowires **thinner than 60 nm** can be printed by applying grayscale masks (Fig. 5(c)), **well-defined woodpiles with porosity** can be printed (Fig. 5(a)), and **stitching artifacts at overlapping fields can be compensated** (Fig. 5(b)). We believe that we have provided sufficient demonstrations here to highlight the nonobvious benefits of our grayscale approach. We intend to follow-up this work with a new paper on printing of micro-optics to highlight the more obvious benefits of GP-TPL.

8. The authors intend to present woodpile structures in relation to their analysis on achievable linewidth and pore size; however, the only clear examples of woodpiles are the fully 3D structures shown in the main text (Fig. 3d) and in the supplementary material (e.g., Fig. S7 and Fig. S17d). The remaining examples more closely resemble 2D grid patterns stacked vertically rather than genuine woodpiles. Moreover, Figure S7a shows that even with grayscale control, proximity effects remain significant, with central regions exhibiting clear over-polymerization. It also appears that woodpile features of comparable quality can be achieved without grayscale control, raising questions about the added value of the proposed approach for this specific structure type.

It is expected that some structures in Fig. S7(a) from our original submission (renumbered as Fig. S8(a) in revised version) will show over-polymerization in the central region due to proximity effects at high exposures. This is because the entire structure was printed with the same motif (i.e., grayscale compensation for proximity effects was not applied for these structures). The goal of this figure was to show that changing the motif provides a deterministic knob to tune the feature size. We have provided additional images of 3D woodpiles that show distinct nanowires in the various layers (Figs. 5(a) and S24). As discussed in response to comment 4, we have now demonstrated printing of woodpiles with higher quality.

9. The authors aim to demonstrate flexibility by using two lenses and state that grayscale-projection TPL can be generalized to various optics. However, switching from a 60x NA 1.3 lens to a 40x NA 1.25 lens mainly affects the field of view; the focusing properties are only marginally different. It would be useful to clarify whether dry objective lenses with NA values close to 1 can also be used.

We want to highlight that although the NA of the two lenses are close, they behave significantly differently within the context of projection-based TPL and GP-TPL due to the large difference in the magnification. The magnification affects not only the field of view but also the pitch of the pixels in the projected image. For example, the pixel pitch of the 60× lens is 114 nm whereas it is 171 nm for the 40× lens. As the NA of the two lenses is similar (i.e., similar focusing properties), 7 pixels fit within the diffraction-limited footprint of the 60× lens but only 5 pixels fit in the footprint of the 40× lens. This major difference means that the motif patterns corresponding to the desired intensity level will change when the lens is changed. For example, motif number 31 for 60× and motif number 16 for 40× reduce the intensity by the same fraction but have different patterns. By identifying the motif sets for the two different lenses, we demonstrated that despite the change in optical configuration, our GP-TPL approach can be used to deterministically tune the intensity of each focal spot. Therefore, the data provided in Fig. 7 strongly supports the generalizability of the GP-TPL approach.

With respect to the specific lens identified by the reviewer: Yes, we believe dry lenses with NA values ~1 will also work for GP-TPL after recalibration of the motif patterns based on the new pixel pitch and the new diffraction-limited spot size.

10. The claim of exceeding 75 million voxels/s and >200 mm3/h with <300 nm features is highly sensitive and requires careful contextualization beside the benchmark analysis provided. Printing rate depends strongly on setup design, resist properties, target geometry, and other parameters. Somers et al. (https://doi.org/10.1038/s41377-021-00645-z) already achieved ~10^8voxels/s using a 100x lens, and the manuscript itself notes that lower-magnification lenses increase throughput. Parallel-scanning DMD-TPL systems have achieved even higher printing rates, ranging from 10^8 voxels/s (https://pubs.acs.org/doi/10.1021/acs.nanolett.4c00505) to 10^12 voxels/s (https://www.light-am.com/article/doi/10.37188/lam.2024.003). Neither of these works is referenced in the manuscript, despite their direct relevance.

We would like to highlight that the reviewer's characterization of the voxel/s rate appears to be incorrect (i.e., higher than the reported value) for two of the studies listed by them. The Somers et al. study reports a rate of 3.2×10^6 voxels/s (not 10^8 voxels/s as claimed by the reviewer) and the performance of this study was already included in our original plots. Additionally, the authors of the last study list a rate of 10^8 voxels/s (not 10^{12} voxels/s as claimed by the reviewer). In the last study (Kiefer et al.), they printed a structure that contained a total of 10^{12} voxels, but the printing was significantly slower at 10^8 voxels/s. Nevertheless, we thank the reviewer for providing the last two references. We have now included these in our comparison plots (Fig. 6). We would also like to highlight that we have now included the definition proposed by Kiefer et al. for the voxel size (as being the demonstrated minimum voxel size) during calculation of voxel generation rate. **With this new definition, our voxel/s rate exceeds 1×10^9 voxels/s.** We have reported both values (original 79 million and revised 1.7 billion voxels/s) in our revised manuscript and provided explanation for these two values. Our voxel/s rate is comparable to or exceeds the state-of-art techniques and our technique still remains the fastest when comparing the volumetric rate in mm^3/hr units. **A key limitation in many of these past studies is that they could not move rapidly from one layer to the next; this reduced their volumetric rate even when their voxel/s rate in each layer was high.** Furthermore, with the additional experiments shown in the revised manuscript, we achieved a minimum **feature width <60 nm, which is the finest ever feature printed under high-speed conditions.** We have appropriately edited the text in main manuscript (paragraph after Fig. 6) to highlight these aspects.

We wholeheartedly agree with the reviewer that the printing rate depends on a variety of factors such as the resist and the optical setup design. If the reviewer's concern is that our superior rate performance is due to a clever selection of lens/resist parameters and not a consequence of the advantages of GP-TPL, this concern can be assuaged by comparing our previous work (study S7 in Fig. 6, Reference 37) with the current work. Both were performed with the same equipment and similar resist, yet GP-TPL enables significantly faster printing. This is because GP-TPL enables rapid printing with a single fs pulse per layer and continuous axial scanning at the repetition rate of the laser. Without GP-TPL, just swapping the lens or changing the resist will not enable this faster printing modality. We have briefly edited the text in the main manuscript (2nd paragraph, page 10) to highlight this enabling capability of GP-TPL. Furthermore, the newly added Fig. S26 highlights that sub-100 nm features can be achieved with GP-TPL by finely controlling the dosage using the motifs. We believe that a more detailed discussion comparing the rate and resolution of this work with each of the past studies listed in Fig. 6 is not necessary to contextualize this work.
